# Transcriptome Analysis of Diffuse Large B-Cell Lymphoma Cells Inducibly Expressing MyD88 L265P Mutation Identifies Upregulated CD44, LGALS3, NFKBIZ, and BATF as Downstream Targets of Oncogenic NF-κB Signaling

**DOI:** 10.3390/ijms24065623

**Published:** 2023-03-15

**Authors:** Marcello Turi, Anjana Anilkumar Sithara, Lucie Hofmanová, David Žihala, Dhwani Radhakrishnan, Alexander Vdovin, Sofija Knápková, Tereza Ševčíková, Zuzana Chyra, Tomáš Jelínek, Michal Šimíček, Annamaria Gullà, Kenneth Carl Anderson, Roman Hájek, Matouš Hrdinka

**Affiliations:** 1 Faculty of Science, University of Ostrava, 70100 Ostrava, Czech Republic; 2 Department of Haematooncology, Faculty of Medicine, University of Ostrava, 70300 Ostrava, Czech Republic; 3 Department of Haematooncology, University Hospital Ostrava, 70800 Ostrava, Czech Republic; 4 Candiolo Cancer Institute, FPO-IRCCS, 10060 Candiolo, Italy; 5 Jerome Lipper Multiple Myeloma Center, LeBow Institute for Myeloma Therapeutics, Dana-Farber Cancer Institute, Boston, MA 02215, USA; 6 Harvard Medical School, Boston, MA 02215, USA

**Keywords:** MYD88, lymphoma, CD44, LGALS3, NF-kB, DLBCL, oncogenic signaling, Galectin-3

## Abstract

During innate immune responses, myeloid differentiation primary response 88 (MyD88) functions as a critical signaling adaptor protein integrating stimuli from toll-like receptors (TLR) and the interleukin-1 receptor (IL-1R) family and translates them into specific cellular outcomes. In B cells, somatic mutations in MyD88 trigger oncogenic NF-κB signaling independent of receptor stimulation, which leads to the development of B-cell malignancies. However, the exact molecular mechanisms and downstream signaling targets remain unresolved. We established an inducible system to introduce MyD88 to lymphoma cell lines and performed transcriptomic analysis (RNA-seq) to identify genes differentially expressed by MyD88 bearing the L265P oncogenic mutation. We show that MyD88^L265P^ activates NF-κB signaling and upregulates genes that might contribute to lymphomagenesis, including CD44, LGALS3 (coding Galectin-3), NFKBIZ (coding IkBƺ), and BATF. Moreover, we demonstrate that CD44 can serve as a marker of the activated B-cell (ABC) subtype of diffuse large B-cell lymphoma (DLBCL) and that CD44 expression is correlated with overall survival in DLBCL patients. Our results shed new light on the downstream outcomes of MyD88^L265P^ oncogenic signaling that might be involved in cellular transformation and provide novel therapeutical targets.

## 1. Introduction

Intracellular adaptor protein myeloid differentiation primary response 88 (MyD88) functions as an indispensable signal transducer for toll-like (TLR) and interleukin-1 (IL-1) family receptors and is thus a critical regulator of innate immunity and inflammation [1]. Upon receptor ligation, MyD88 interacts with activated receptors via the Toll-IL-1-receptor-resistance (TIR) domain and organizes a multiprotein oligomeric signaling complex, the Myddosome [2,3]. The core components of the Myddosome include interleukin-1 receptor-associated kinase (IRAK) family kinases directly interacting with MyD88 with homotypic death domain (DD) interactions [1,4]. MyD88 and IRAK further cooperate with additional signaling proteins, including E3 ubiquitin ligases (e.g., TRAF6, Pellino, and LUBAC), deubiquitinases (e.g., TNFAIP3 and CYLD), and kinases (e.g., TAK1, NEMO/IKK, BTK, and HCK), to activate transcription factor (TF) nuclear factor-κB (NF-κB) and induce the expression of chemokines and other proinflammatory genes [5,6,7,8,9,10].

Lymphoma cells addicted to constitutive pro-survival NF-kB signaling are driven by somatic mutations in genes that aberrantly activate various signaling pathways, such as B-cell receptor (BCR) and members of the CARD11-BCL10-MALT1 (CBM) complex [11,12]. Since MyD88 is the key adaptor protein shared by many diverse receptors, MyD88-dependent signaling is especially prone to deregulation in lymphomagenesis [13,14,15]. Strikingly, a gain-of-function oncogenic somatic mutation in MyD88 is causative of aggressive hematologic malignancies [7,16,17,18], including activated B-cell (ABC) diffuse large B-cell lymphoma (DLBCL; ~39% of total cases) [4], Waldenstrom’s macroglobulinemia (WM; ~95% of total cases), and primary central nervous system (CNS) lymphoma [8]. The recurrent positive dominant mutation L265P (~29% of ABC DLBCL cases) localized in the TIR domain of MyD88 (MyD88^L265P^) leads to the spontaneous oligomerization of MyD88 and drives the activation of NF-kB signaling without any additional stimuli, likely following the above-described phosphorylation and ubiquitination cascade for the formation of the natural Myddosome [4,19,20]. The formal confirmation that the MyD88^L265P^ mutation drives lymphomagenesis came from the study of MyD88^L265P^ mice, which develop a DLBCL-like disease [21,22,23,24]. However, the mutation alone might be insufficient to drive malignant transformation in B cells, and other cooperating factors (such as TLR7/9, BCL2, BTK, and BCR) are likely required for the full development of lymphoma [23,24,25,26,27,28].

Over the last decade, several genomic studies aimed to identify MyD88^L265P^ lymphoma-associated genetic lesions and describe the transcriptomic profiles of malignant cells [26,29,30,31,32]. Despite intensive research, however, the immediate consequences of MyD88^L265P^ expression in lymphoma cells, the exact molecular mechanism of MyD88 oncogenic signaling, and its role in other pathways beyond NF-kB activation remain unresolved. Here, we report the first detailed investigation of acute MyD88^L265P^ expression in lymphoma cell lines. We examine the transcriptomic response of model lymphoma cell lines acutely expressing MyD88^L265P^ and identify downregulated and upregulated downstream targets of MyD88^L265P^ oncogenic signaling that might be involved in cellular transformation.

## 2. Results

### 2.1. Lentiviral Inducible System Makes Acute MyD88^L265P^ Expression Possible in Lymphoma Cells to Study Early Events of NF-κB-Mediated Cell Transformation

**Figure 1:**
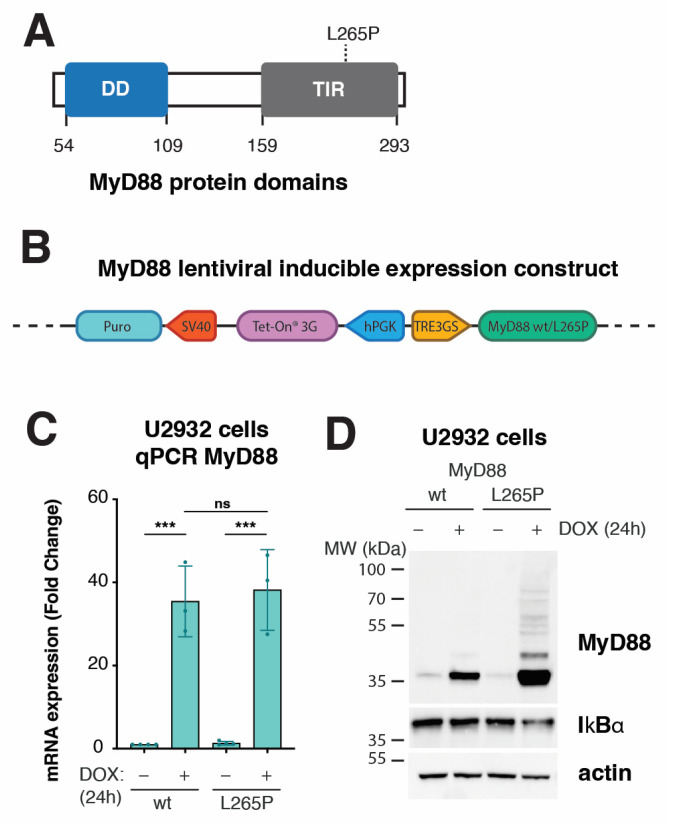
Development of inducible system to express MyD88 wt or L265P in lymphoma cell lines. (**A**) Schematic representation of MyD88 protein domains. The missense mutation (L265P) is located at the TIR domain at the C terminus. (**B**) Schematic representation of the tetracycline-inducible lentiviral gene expression system, pLVX-TetOne-Puro, used for the overexpression of MyD88 (wt/L265P) in stable cell lines. The Tet-On 3G transactivator is constitutively expressed under the human PGK promoter. The MyD88 gene (NM_002468.5) is under the TRE3GS promoter in the opposite orientation. In the presence of doxycycline (DOX), the Tet-On 3G transactivator binds and activates the TRE3GS inducible promoter that controls MyD88 expression. The gene sequence encoding cell selection marker puromycin N-acetyltransferase (Puro) under the simian virus 40 (SV40) promoter confers puromycin resistance. (**C**) MyD88 inducible expression analyzed with qPCR after 24 h of DOX (250 ng/mL) treatment in U2932 cell lines (ns, not significant for *p* > 0.05, *** for *p* ≤ 0.001). (**D**) Western blot analysis of MyD88 inducible expression after 24 h of DOX (250 ng/mL) treatment in U2932 cell lines. IkBα staining was used as a marker for NF-kB pathway activation. Actin was used as a loading control.

It has been established that MyD88^L265P^ triggers oncogenic NF-κB signaling independent of receptor stimulation [4,20,33]. Searching for genes and pathways deregulated by MyD88^L265P^ in lymphoma, we developed model cell lines with inducible expression of wildtype (wt) or mutant MyD88^L265P^ variants. Briefly, we cloned MyD88 (isoform NM_002468.5 and its mutated version L265P) (Figure 1A) into lentiviral Tet-On-3G inducible vector pLVX-TetOne-Puro [34] (Figure 1B and Figure S1A), which offers tight and dynamic control using doxycycline (DOX) without leaky transgene expression. First, we extensively tested our MyD88 inducible system with the THP1-Dual^TM^ monocyte cell line (Invivogen, San Diego, CA, USA), which can simultaneously monitor the activation of both NF-κB and interferon regulatory factor (IRF) signaling pathways by assessing the activity of reporter proteins secreted alkaline phosphatase (SEAP) and secreted luciferase (Lucia), respectively. As a result of increasing DOX concentration to induce the expression of MyD88^L265P^, we could detect significantly increased activity of the NF-κB reporter (Figure S1B). Interestingly, upon MyD88^L265P^ expression, we could also observe increased activation of the IRF reporter, albeit reporter activation first appeared at 100 ng/mL DOX (Figure S1C). In contrast, the expression of wt MyD88 failed to induce any significant IRF reporter activation and only subtle (1.9-fold) NF-κB reporter activation (Figure S1B,C), which is consistent with previous reports [35,36,37]. Subsequent Western blotting (WB) analysis confirmed the results of the NF-κB reporter assay, as we could only detect increased levels of phosphorylated NF-κB subunit p65 (p-p65) and decreased total levels of inhibitor of NF-kB alpha (IkBα) in the cell extracts of MyD88^L265P^-expressing cells (Figure S1D).

As a lymphoma model, we chose the ABC DLBCL cell line U2932 endogenously expressing low levels of wt MyD88 [32]. First, we tested transduced U2932 cells for inducible MyD88 expression at both transcript and protein levels. Upon DOX treatment, qPCR analysis revealed about 35-fold MyD88 mRNA upregulation of both MyD88 wt and L265P compared with uninduced cells (Figure 1C). Moreover, Western blotting for total levels of IkBα confirmed active NF-κB signaling, specifically in DOX-induced MyD88^L265P^ cells (Figure 1D). Interestingly, we could consistently detect increased total protein levels of MyD88^L265P^, suggesting higher protein stability conferred by the L265P mutation. This observation agrees with reports showing that MyD88^L265P^ forms a much more stable Myddosome [20,38,39]. In addition, immunoblotting for MyD88 revealed higher MW entities corresponding to polyubiquitinated MyD88 [40,41,42,43] (Figure 1D).

### 2.2. Transcriptome Analysis (RNA-Seq) of Genes Differentially Expressed in U2932 Lymphoma Cells Acutely Expressing MyD88^L265P^

**Figure 2:**
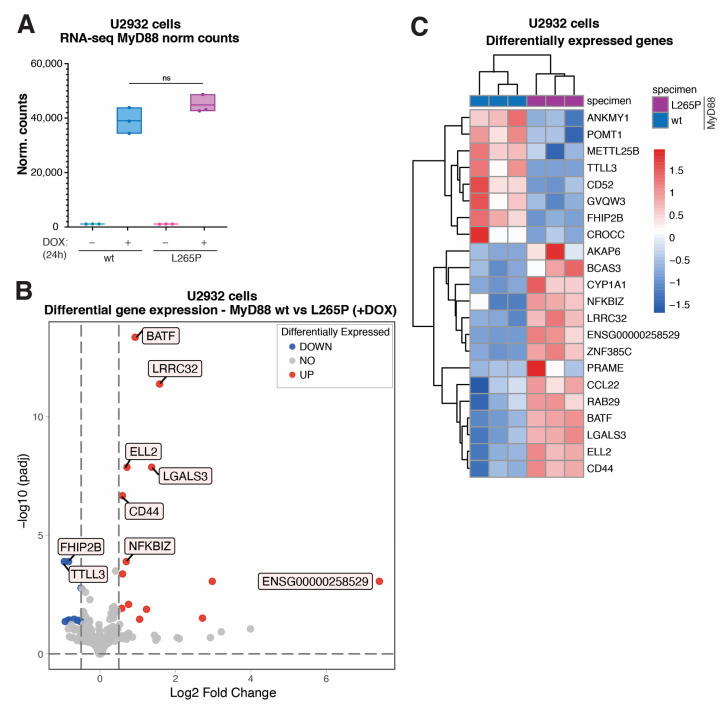
Transcriptome analysis (RNA-seq) of genes differentially expressed in U2932 lymphoma cells acutely expressing MyD88^L265P^. (**A**) MyD88 normalized counts resulted from transcriptome analysis (ns for *p* > 0.05). (**B**) Volcano plot showing differentially expressed genes in U2932 cell lines inducibly expressing MyD88 vs. MyD88^L265P^. The blue dots indicate downregulated genes, and the red dots indicate upregulated genes with Benjamini and Hochberg corrected *p*-value less than 0.05 and an absolute value of log2 fold change greater than 0.5. (**C**) Heatmap representation of all the significantly differentially expressed genes (as described in (**B**)) in U2932 cell lines expressing MyD88 wt vs. L265P.

Next, having established and validated the MyD88 inducible system in lymphoma cells, we wanted to reveal the global effects of acute MyD88^L265P^ expression at the transcriptome level. To this end, we isolated total RNA from DOX-induced (+DOX) U2932 cells expressing MyD88 and MyD88^L265P^, and uninduced controls (–DOX); generated a TruSeq stranded mRNA library; and performed RNA-seq analysis using a NovaSeq 6000 Illumina platform. The expression of both MyD88 (Figure S2B–D, Tables S1 and S2) and MyD88^L265P^ (Figure S2B–F, Tables S1 and S2) resulted in gene expression changes. However, as expected and in accord with upregulated signaling pathways (Figure 1 and Figure S1), we identified substantially more differentially expressed genes in MyD88^L265P^- vs. MyD88-expressing cells (97 vs. 15 genes; log2 fold change > 0.5, adj. *p*-value ≤ 0.05). Importantly, the normalized counts of MyD88 transcripts in our RNA-seq datasets confirmed similar levels of MyD88 and MyD88^L265P^ (Figure 2A), which agrees with our qPCR results (Figure 1C). Subsequent differential gene expression analysis of MyD88 vs. MyD88^L265P^ samples identified the eight most significantly downregulated genes (ANKMY1, POMT1, METTL25B, TTLL3, CD52, GVQW3, FHIP2B, and CROCC) and fourteen upregulated genes (AKAP6, BCAS3, CYP1A1, NFKBIZ, LRRC32, ENSG00000258529, ZNF385C, PRAME, CCL22, RAB29, BATF, LGALS3, ELL2, and CD44) in cells expressing MyD88^L265P^ (Figure 2B,C and Figure S2A, Tables S1 and S2).

### 2.3. Validation of Top Upregulated Genes Identified with RNA-Seq Analysis Using Public Expression Datasets and with qPCR and Western Blotting

**Figure 3:**
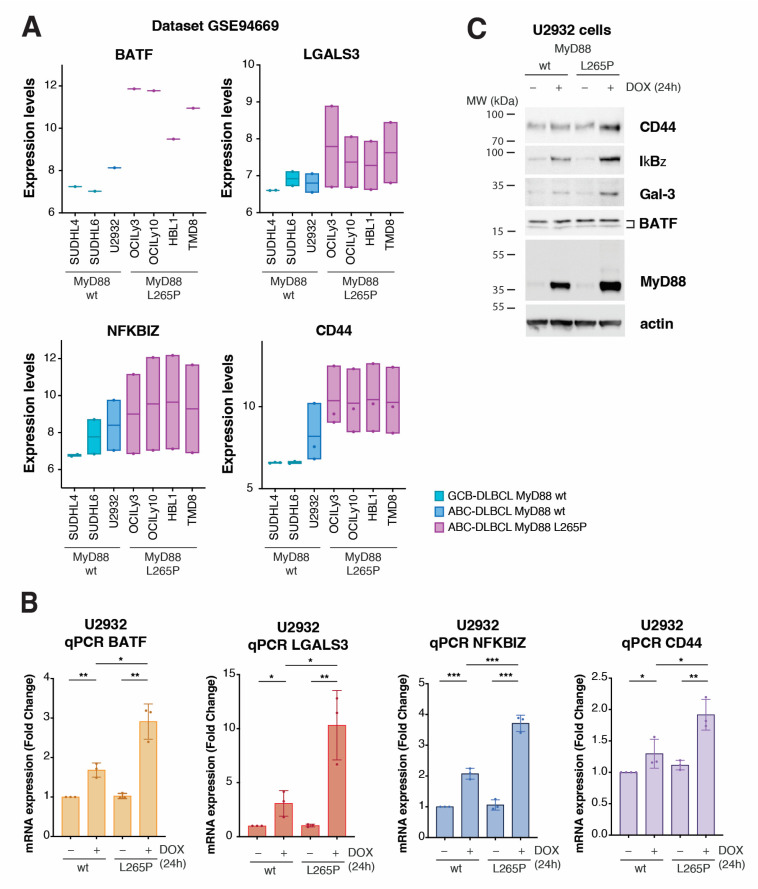
Validation of transcriptomic results using publicly available datasets and in the model cell lines. (**A**) Gene expression validation in the immortalized cell lines, using microarray expression dataset GSE94669 for genes *BATF*, *LGALS3*, *NFKBIZ,* and *CD44*. In the dataset containing the gene expression of 61 cell lines, 7 representative cell lines were considered. Cell lines SUDHL4 and SUDHL6 represent GCB DLBCL with wt MyD88. U2932 represents ABC DLBCL with wt MyD88. OCI-Ly3, OCI-Ly10, HBL1, and TMD8 represent ABC DLBCL with MyD88^L265P^. The *y*-axis represents the normalized expression according to microarray data. (**B**) qPCR validation of differentially expressed genes *BATF*, *LGALS3*, *NFKBIZ,* and *CD44* in the U2932 cell line upon MyD88 (wt/L265P) inducible expression after 24 h of DOX treatment (* for *p* ≤ 0.05, ** for *p* ≤ 0.01, *** for *p* ≤ 0.001). (**C**) Western blot validation of the 4 hits (BATF, Gal-3, IkBƺ, and CD44) upon MyD88 (wt/L265P) inducible expression after 24 h of DOX treatment (250 ng/mL) in the U2932 cell line. Actin was used as a loading control.

We next sought to validate the top upregulated genes from our RNA-seq results using independent gene expression datasets and experimental methods. First, we wondered whether any MyD88^L265P^-upregulated gene exhibited a specific expression pattern in DLBCL cell lines. To this end, we analyzed the publicly available gene expression profiles of 61 lymphoma cell lines obtained using Illumina HumanHT-12 V4.0 expression BeadChip GSE94669 [32]. The comparison of gene expression levels in germinal center B-cell-like (GCB) DLBCL (SUDHL6) and ABC DLBCL cell lines with MyD88 (SUDHL4 and U2932) vs. MyD88^L265P^ ABC DLBCL (OCI-Ly3, OCI-Ly10, HBL1, and TMD8) revealed consistently higher expression of genes BATF, LGALS3, NFKBZ, and CD44 in cells bearing the MyD88 ^L265P^ mutation (Figure 3A), which was not the case for the other most significantly upregulated genes (Figure S3A). In the same comparison, the two most significantly downregulated genes, TTLL3 and FHIP2B, did not show similar deregulation in the dataset used (Figure S3A). The putative gene ENSG00000258529, provisionally annotated based on gene homology, resulting to be a mannosyltransferase, was not present in the GSE94669 dataset. For these reasons, we focused on further validating genes BATF, LGALS3, NFKBZ, and CD44. Using U2932 cells with MyD88 inducible expression, we could confirm significantly upregulated mRNA for BATF, LGALS3, NFKBZ, and CD44 using qPCR on MyD88^L265P^-expressing cells (Figure 3B). We also conducted qPCR for ELL2 and RAB29, since they were upregulated in 75% of the cell lines with the MyD88^L265P^ mutation. According to the RNA-seq data, while ELL2 showed higher significance (Figure S2A, Table S1), qPCR also confirmed this. However, RAB29 did not show significant upregulation in qPCR (Figure S3B). Additionally, we aimed to validate the two most downregulated genes, TTLL3 and FHIP2B, but we could not observe significant downregulation of these genes in U2932 cells with MyD88 ^L265P^ inducible expression using qPCR. Since increased expression levels might not always translate into more abundant proteins, we also performed a Western blotting analysis of cell extracts obtained from inducible MyD88 U2932 cell lines to check the total protein levels of BATF, LGALS3 (Galectin-3 and Gal-3), NFKBZ (IkBƺ), and CD44 (Figure 3C). While CD44, Gal-3, and IkBƺ levels were significantly increased in MyD88^L265P^-expressing cells, we did not see any change in the protein levels of BATF, suggesting the possible complex regulation of BATF translation or turnover [44]. Our qPCR and WB results confirm the previously reported IkBƺ upregulation by MyD88^L265P^ in U2932 cells [45].

### 2.4. CD44 Is a Downstream Target of Oncogenic NF-κB Signaling in MyD88^L265P^-Expressing Lymphoma Cells

**Figure 4:**
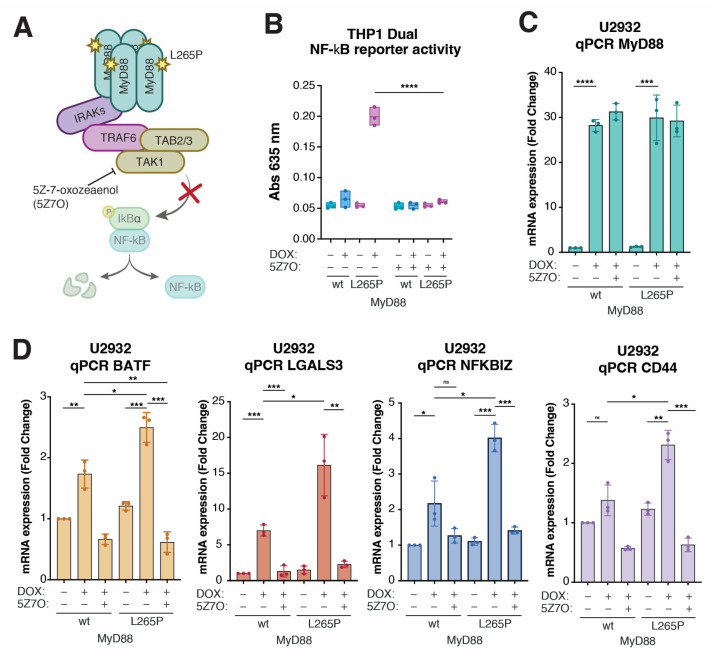
CD44 is a downstream target of NF-kB signaling in MyD88^L265P^-expressing lymphoma cells. (**A**) Schematic representation of the signaling pathway triggered by the autodimerization of MyD88^L265P^ and the role of TAK1 inhibitor 5Z-7-oxozeaenol (5Z7O) in the pathway. (**B**) Validation of the TAK1 inhibitor 5Z-7-oxozeaenol (5Z7O) in THP1 Dual cell lines. Cells were treated with 1 µM 5Z7O and 250 ng/mL DOX as indicated. After 24 h, MyD88- and MyD88^L265P^-induced NF-kB activation was measured with the QUANTI-Blue method. (**C**) qPCR analysis of MyD88 expression levels in U2932 cell lines after 24 h DOX and subsequent 24 h 5Z7O (1 µM) treatments. (**D**) qPCR analysis of expression levels of BATF, LGALS3, NFKBIZ, and CD44 in U2932 cell lines after 24 h DOX (250 ng/mL) and subsequent 24 h 5Z7O (1 µM) treatments. (ns, not significant for *p* > 0.05, * for *p* ≤ 0.05, ** for *p* ≤ 0.01, *** for *p* ≤ 0.001, **** for *p* ≤ 0.0001).

The critical step in NF-κB activation downstream of MyD88 signaling involves transforming growth factor beta-activated kinase 1 (TAK1; MAP3K7) [46] (Figure 4A). We could demonstrate that the selective TAK1 inhibitor 5Z-7-oxozeaenol (5Z7O) [47] completely abolished the MyD88^L265P^-induced upregulation of NF-κB reporter activity in THP1 Dual cells (Figure 4B) without affecting the DOX-induced expression of MyD88 or MyD88^L265P^ (Figure 4C). Thus, we hypothesized that TAK1 inhibitor 5Z7O could prevent the upregulation of BATF, LGALS3, NFKBZ, and CD44 in MyD88^L265P^ U2932 cells if they were NF-κB targets. Indeed, the qPCR analysis of U2932 cells induced to express MyD88 or MyD88^L265P^ revealed that 5Z7O effectively blocked MyD88-induced BATF, LGALS3, NFKBZ, and CD44 expression (Figure 4D), suggesting that these genes are under NF-κB transcriptional control. We also conducted a WB analysis of cell extracts from inducible MyD88 U2932 cell lines, treated as for qPCR, with TAK1 inhibitor 5Z7O, to check the total protein levels of BATF, Gal-3, IkBƺ, and CD44. The analysis confirmed the same pattern observed in the qPCR experiment (Figure S4A).

### 2.5. CD44 Surface Levels Are Correlated with NF-κB-Activating MyD88^L265P^ Expression, and CD44 Expression Stratifies DLBCL Subsets and Predicts Overall Survival in DLBCL Patients

**Figure 5:**
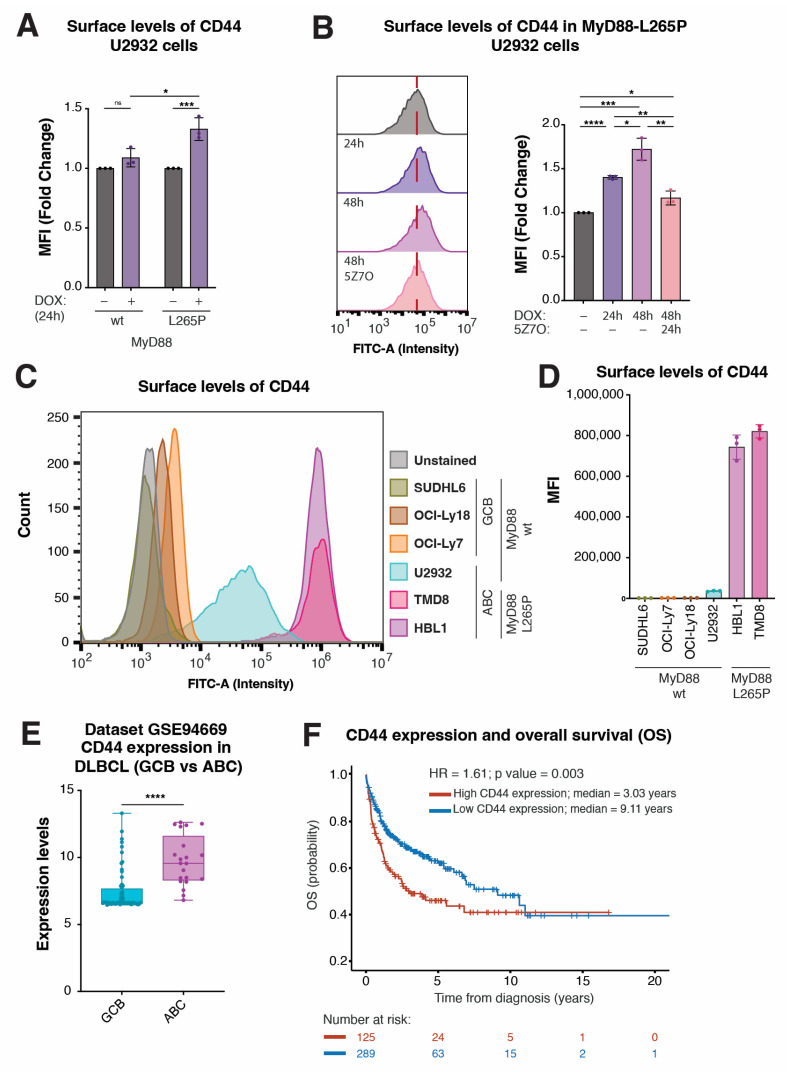
CD44 levels are correlated with MyD88 L265P expression and predict OS in DLBCL patients. (**A**) Mean fluorescence intensity (MFI) of CD44-FITC staining obtained with flow cytometry analysis of U2932 cell lines upon MyD88 (wt/L265P) inducible expression after 24 h of DOX treatment. (**B**) Histogram and related MFI of FITC obtained with flow cytometry analysis of U2932 cell lines upon MyD88 (wt/L265P) inducible expression after DOX and subsequent 5Z7O (1 µM) treatments for the time intervals annotated in the figure. (**C**) Histogram showing the difference in CD44 surface levels, analyzed with surface staining and flow cytometry in SUDHL6, OCI-Ly18, and OCI-Ly7 cell lines (which represent GCB DLBCL with wt MyD88). The U2932 cell line represents ABC-DLBCL with wt MyD88. HBL1 and TMD8 represent ABC DLBCL with MyD88^L265P^. (**D**) Flow cytometry analysis of SUDHL6, OCI-Ly18, OCI-Ly7, U2932, HBL1, and TMD8 cell lines stained with CD44-FITC-conjugated antibody shown as MFI. (**E**) Validation of CD44 expression levels in the subgroups of GCB and ABCL DLBCL cell lines from the GSE94669 dataset. (**F**) Kaplan–Meier curves for OS in DLBCL patients using the transcriptome profiling and clinical information of 449 DLBCL patients from GEO dataset (GSE10846; n = 420). The average expression of 13 CD44 probes for each patient was used for overall representation of clinical data. The patients were divided into two groups based on the CD44 expression level. High-CD44 (red) and low-CD44 (blue) groups were determined as described in the Section 4. (ns, not significant for *p* > 0.05, * for *p* ≤ 0.05, ** for *p* ≤ 0.01, *** for *p* ≤ 0.001, **** for *p* ≤ 0.0001).

Compared with genes BATF, LGALS3, and NFKBIZ, the role of CD44 in DLBCL is less clear. Thus, we explored the regulation of CD44 expression by MyD88^L265P^ in more detail. Many previous studies reported an important functional role of CD44 in various types of cancer [48,49,50,51,52,53,54] and increased CD44 expression has also been observed in lymphoma [55,56,57,58,59,60]. Since we identified CD44 as a prominent downstream target of MyD88^L265P^ oncogenic NF-κB signaling, we wondered whether CD44 might serve as a cell surface marker of MyD88^L265P^-dependent DLBCL lymphoma. The flow cytometry analysis of cells inducibly expressing MyD88^L265P^ for 24 h revealed increased surface CD44 levels in U2932 lymphoma cells (Figure 5A) as well as other cell types, such as THP1 and U2OS (Figure S5A), which could be reverted with 5Z7O treatment (Figure 5B). Using flow cytometry, we further evaluated CD44 surface levels on a small panel of DLBCL lymphoma cell lines of GCB (SUDHL6, OCI-Ly18, and OCI-Ly7) and ABC (U2932, TMD8, and HBL1) origin (Figure 5C,D). Interestingly, all ABC DLBCL cell lines showed increased CD44 staining compared with GCB DLBCL cell lines; however, the two ABC DLBCL cell lines bearing MyD88^L265P^ (TMD8 and HBL1) expressed the highest levels of CD44 (Figure 5C,D).

Next, we analyzed the GSE94669 expression dataset of human lymphoma cells to validate these observations on a larger sample. Comparing the CD44 expression levels in GCB (20 cell lines and three probes) vs. ABC (7 cell lines and three probes) DLBCL cell lines, we found significantly (1.3-fold) increased CD44 expression in the ABC DLBCL samples (Figure 5E). Finally, we wondered whether there might be clinical relevance to high CD44 expression in DLBCL patients. To this end, we analyzed the transcriptome profiling and clinical information of 420 DLBCL patients from GEO dataset GSE10846 [61,62]. Upon dividing the samples into two groups based on the CD44 expression level (high vs. low; 13 probes), Kaplan–Meier analysis revealed significantly worsened overall survival (OS) probability in CD44-high DLBCL cases (Figure 5F and Figure S5B). Importantly, we confirmed high CD44 expression to be an independent prognostic factor when combined with age, sex, and treatment in multivariate Cox analysis (HR = 1.61, *p* = 0.004) (Supplementary Table S3). Thus, CD44 might serve as a marker of MyD88^L265P^-dependent ABC DLBCL and potentially as a novel, valuable prognostic factor.

## 3. Discussion

In this study, we aimed to uncover the early transcriptomic response of MyD88^L265P^ using a newly established, tightly controlled model lymphoma cell line. The main reason for choosing an inducible expression system over constitutive expression was to avoid the negative feedback loop known to regulate NF-κB signaling and the potential adaptation of lymphoma cells to chronic MyD88^L265P^ expression. NF-κB signaling is tightly controlled [63,64], and MyD88^L265P^-induced NF-κB signaling triggers a negative feedback loop in humans [43,65] and mice [21] that operates on several levels, including deubiquitinase A20 (TNFAIP3)- and Bim-dependent apoptosis [21,43,65]. Additionally, the lentiviral delivery of this system followed by cell selection (puromycin or GFP sorting) resulted in a more homogenous genetically modified cell population than transient overexpression using electroporation (not shown). In our approach, we induced MyD88^L265P^ expression with a titrated amount of DOX for 24 h, which was sufficient to drive a strong NF-κB transcriptomic response in all cell lines tested (THP1 Dual, U2OS, and U2932). The observed discrepancy between MyD88^L265P^ mRNA and protein levels might have been due to enhanced MyD88^L265P^ protein stability in oligomeric form [38,39], or might have been cell-type specific, and warrants future investigation.

Interestingly, high MyD88 protein levels irrespective of mutation status in DLBCL are associated with tumor recurrence and shortened survival in patients [66]. Thus, to avoid the potential effects of increased endogenous MyD88 expression in our experimental model, we chose the ABC DLBCL cell line U2932, endogenously expressing low amounts of MyD88 wt [32]. Previous studies have reported transcriptomic profiles of primary lymphoma cells bearing MyD88 mutations [26,30,31,67]; however, to the best of our knowledge, this is the first study solely addressing the transcriptomic response of MyD88^L265P^ in a well-characterized and tightly controlled cellular model system. Therefore, our system is a highly informative model for transcriptomic studies, and the obtained results might reflect early cellular transformation events in MyD88^L265P^-triggered lymphomagenesis.

As expected, the transcriptomic analysis (RNA-seq) revealed substantial gene expression changes in DOX-induced MyD88^L265P^ compared with uninduced or MyD88-expressing U2932 cells. Furthermore, differential gene expression analysis of cells inducibly expressing MyD88 vs. MyD88^L265P^ revealed gene sets specifically deregulated by the MyD88^L265P^ mutation. Amongst the eight most significantly downregulated genes, the molecular functions of genes ANKMY1, METTL25B, and GVQW3 remain largely uncharacterized. The biological functions of FHIP2B, POMT1, TTLL3, and CROCC have been reported in the literature and are summarized in Supplementary Table S4. CD52 is a GPI-linked membrane protein mainly expressed in lymphocytes. The function of CD52 is not well characterized, but it is thought to regulate immune responses and might play a role in cancer development [68]. High levels of CD52 expression have been observed in lymphoma and leukemia, and CD52 may play a role in the growth and survival of cancer cells [69,70]. Immunotherapeutic monoclonal antibody Alemtuzumab targets CD52 and is used in chronic lymphocytic leukemia (CLL) therapy [69,71]. However, the exact roles and specific functions of FHIP2B, POMT1, TTLL3, CROCC, and CD52 in lymphoma and the molecular mechanisms of their downmodulation upon MyD88^L265P^ expression are not yet fully understood and require further research.

Amongst the fourteen most significantly upregulated genes upon MyD88^L265P^ expression, we identified ENSG00000258529, provisionally annotated as a mannosyltransferase with a completely unknown function. Two other genes, ZNF385C and PRAME, were previously reported to be overexpressed in specific cancer types but with largely unknown molecular functions (Supplementary Table S4). PRAME is highly expressed in cancer, including hematological malignancies [72,73,74], and as a tumor-associated antigen (TAA), it represents a potential immunotherapy target [75,76,77]. The function of PRAME might be cancer-type specific [78]. In DLBCL, PRAME was found to interact with the EZH2 protein, and PRAME deletions were associated with poor outcomes [79]. More research is needed to understand the function of these genes in cancerogenesis and their potential roles in MyD88^L265P^-driven lymphoma.

We also identified seven genes (ELL2, CYP1A1, RAB29, AKAP6, BCAS3, LRRC32, and CCL22) involved in general cellular processes and known molecular functions (transcription, metabolism, trafficking, and signaling) but with an unclear link with MyD88^L265P^ and lymphomagenesis (Supplementary Table S4). For instance, the eleven nineteen lysine-rich leukemia 2 (ELL2) gene, which encodes an elongation factor for RNA polymerase II, is involved in antibody secretion, unfolded protein response, and plasma cell development [80,81,82]. In ABC DLBCL, EEL2 represents one of the enrichment signature genes [83]. Leucine-rich repeat containing 32 (LRRC32), also known as glycoprotein A repetitions predominant (GARP), is a vital membrane receptor involved in the activation of immunosuppressive cytokine TGF-β in immune cells, including T regs, platelets, and B cells activated via TLRs [84,85,86,87]. High LRRC32 expression is associated with immune evasion, increased cancer cell proliferation, and survival [86,87,88,89] and represents an emerging target for cancer immunotherapy [90]. Chemokine CCL22 (also known as macrophage-derived chemokine; MDC) is produced by various cell types, including B cells and cancer cells [91]. Several studies have demonstrated CCL22 involvement in maintaining a suppressive tumor microenvironment, and the development and progression of cancer, including lymphoma [92,93,94,95]. In DLBCL, CCL22 has been described in the gene enrichment signature [83,96]. However, the exact roles and molecular functions of ELL2, CYP1A1, RAB29, AKAP6, BCAS3, LRRC32, and CCL22 in lymphomagenesis are not fully understood. More research is needed to determine whether targeting these proteins could be a potential therapeutic approach for treating cancer.

Based on public gene expression profiles and literature searches for reported functions, we selected four upregulated genes for validation. Two of those genes, BATF and NFKBIZ, are well-known transcriptional regulators. The top hit identified in our RNA-seq, basic leucine zipper ATF-like TF (BATF), a member of the activator protein 1 (AP-1)/ATF superfamily of TF, plays a key role in the modulation of the AP-1 transcription complex [97], particularly in immune cells such as T cells and B cells [98,99,100]. To exert its regulatory function, BATF forms complexes with several members of the interferon-regulatory factor (IRF) family and other AP-1 TF [97,101,102]. BATF is involved in the development and function of immune cells, and the activation of immune responses [98,99,100,102,103,104]. According to some studies, BATF may play a role in the development and progression of certain types of cancer, including leukemia and lymphoma [101,105,106]. High BATF expression was demonstrated in DLBCL samples [106] and is considered a part of the gene enrichment signature of ABC DLBCL [83,107]. Here, we identified BATF as a top upregulated gene at the mRNA level in lymphoma cells inducibly expressing MyD88^L265P^. Interestingly, according to the Harmonizome database, BATF might contribute to the transcriptional regulation of two other upregulated genes in our dataset, EEL2 and NFKBIZ [108]. However, more research is needed to understand BATF regulation and function in lymphoma.

Similarly, nuclear factor of kappa light polypeptide gene enhancer in B cells inhibitor zeta (NFKBIZ), a member of the nuclear I-kappa-B family, stabilizes the promoter binding of other transcription regulators and is involved in the transcriptional control of inflammation, cell proliferation, and survival [109,110,111]. Depending on the context, the IκBζ protein can promote or inhibit gene expression and the activation of signaling pathways involved in producing inflammatory molecules [110,112]. Several studies have suggested that NFKBIZ may be a driver gene for the development and progression of certain types of cancer, including lymphoma [110,113]. High IκBζ expression was explicitly detected in ABC DLBCL [45]. Moreover, the amplification of the NFKBIZ locus has been observed in ~10% of ABC DLBCL cases [114], and NFKBIZ mutations affecting 3’UTR can stabilize the NFKBIZ transcript and lead to the overexpression of the IκBζ protein, which activates the NF-κB signaling pathway and provides a selective advantage to tumor cells [115,116]. In normal B cells, NFKBIZ expression is induced by BCR or TLR stimulation [117] and an increase in the NFKBIZ transcript and IκBζ protein was demonstrated due to constitutive oncogenic NF-κB signaling in MyD88^L265P^- or CARD11^L244P^-expressing lymphoma cells [45]. Here, we independently confirmed high NFKBIZ expression in MyD88^L265P^ lymphoma cells and identified NFKBIZ as one of the top genes upregulated by MyD88^L265P^ oncogenic NF-κB signaling. Due to the addiction of ABC DLBCL to NFKBIZ expression, IκBζ might represent a promising therapeutic target for drug development [45].

Two other genes, LGALS3 and CD44, encode very well-known transmembrane receptors galectin-3 (Gal-3) and glycoprotein cluster of differentiation 44 (CD44), respectively. Gal-3 is a multifunctional member of the galectin protein family with an affinity for beta-galactosides (such as N-acetyllactosamine) and advanced glycosylation end (AGE) products [118] and is involved in a variety of cellular processes, including cell adhesion, proliferation, differentiation, apoptosis, signaling, and immune system function [119,120]. Gal-3 regulates many complex interactions of cells within the tumor microenvironment and has been studied for its role in the cancer development, progression, and maintenance of cancer stem cells [121,122,123]. For instance, in acute myeloid leukemia (AML), chronic lymphocytic leukemia (CLL), and classical Hodgkin’s lymphoma (cHL), Gal-3 overexpression is associated with poor survival and prognosis [124,125,126]. Similarly, in DLBCL, multiple studies associated overexpressed Gal-3 with increased cell proliferation, survival, and disease aggressiveness and identified GAL-3 as a prognostic factor and potential target for therapy [127,128,129,130,131,132,133]. Interestingly, Gal-3 binds lymphocyte-activation gene 3 (LAG3), the immune checkpoint of immune effector cells, and anti-LAG-3 (relatlimab) represents a novel FDA-approved inhibitor for combinational checkpoint therapy in melanoma [134]. Likely, targeting the LAG3/Gal-3 axis could also be beneficial to therapy for other malignancies [135,136]. Our present study further links Gal-3 overexpression in DLBCL with MyD88^L265P^ oncogenic signaling.

CD44 is expressed on the surface of many cell types, including immune cells such as T cells and B cells, and is the most common cancer stem cell (CSC) marker in multiple types of cancers [137,138]. It is a multifunctional transmembrane receptor binding to various ligands, including hyaluronic acid (HA), collagen, and osteopontin, which can modulate its activity [139]. CD44 has multiple, functionally diverse isoforms generated by the alternative splicing of the CD44 gene [140,141]. CD44 plays a role in cell adhesion and migration, and it is involved in the activation and regulation of the immune system, and the formation and maintenance of the extracellular matrix [139,142]. Some studies have suggested that CD44 may play a role in the development and progression of lymphoma. For example, overexpressed CD44 in lymphoma cells [55,59,60] is associated with increased cell proliferation and survival [54]. In particular, cells expressing CD44 showed elevated levels of local tumor formation, correlated with aggressive metastatic behavior [60]. Since CD44 promotes the mobilization of anti-apoptotic mechanisms, it seems to play a negative role in hematological diseases [143]. In ABC DLBCL, CD44 was identified as a part of the gene enrichment signature [83]. Additionally, targeting CD44 with specific drugs has been shown to inhibit the growth and proliferation of lymphoma cells in culture and animal models [144,145,146].

Interestingly, analyzing the publicly available dataset GSE94669, we found that out of the most significant MyD88^L265P^-upregulated genes, CD44, BATF, LGALS3, and NFKBIZ exhibited an expression pattern specific to ABC DLBCL cell lines, with the highest expression in ABC DLBCL cell lines bearing the L265P mutation. We also identified the same expression pattern in a mouse lymphoma model of mutant MyD88 (Figure S6; dataset GSE141453 [147]) and other available lymphoma datasets (not shown; e.g., GSE50721 [148], GSE56315 [149,150], and GSE31312 [151]). Since literature evidence supports the important roles of CD44, BATF, LGALS3, and NFKBIZ in cancer, we decided to validate the expression of these genes with independent experimental methods. Using qPCR, we demonstrated that all four genes are significantly upregulated at the mRNA level in MyD88^L265P^-expressing cells. Moreover, we could also detect evident upregulation at the protein level, apart from BATF. Why increased BATF transcription does not translate into more abundant BATF protein in our experimental settings is currently unclear and remains to be addressed in the future.

Since MyD88^L265P^ oncogenic signaling in lymphoma cells leads to the activation of the NF-κB pathway, we hypothesized that the inhibition of NF-κB signaling could prevent the observed transcriptional changes in MyD88^L265P^-expressing cells. Indeed, blocking the NF-κB pathway with a selective inhibitor at the level of TAK1 completely blocked the MyD88^L265P^-induced expression of CD44, BATF, LGALS3, and NFKBIZ. Moreover, while the presence of NF-κB binding sites in the promoters of CD44, LGALS3, and NFKBIZ is well documented, NF-κB binding sited in the BATF promoter has not been reported [152]. Thus, we cannot exclude the possibility that BATF upregulation by MyD88^L265P^ is indirect and secondary to the activation of the NF-κB signaling pathway.

Analyzing the publicly available dataset GSE94669, we could also notice that even though CD44 is a known marker for ABC DLBCL classification [83], CD44 levels are consistently higher in lymphoma cell lines expressing mutated MyD88. To experimentally confirm this observation, we measured CD44 levels using flow cytometry in a panel of six DLBCL cell lines and three inducible MyD88^L265P^ cell lines. Consistently with gene expression analysis, the surface CD44 levels were significantly higher in cells where MyD88^L265P^ was either constitutively or inducibly expressed. Even though CD44 is one of the NF-kB signature genes and a known marker for the discrimination between GCB and ABC DLBCL [153], this is the first report of MyD88^L265P^ directly enhancing the expression and surface levels of CD44. Moreover, our analysis of GEO dataset GSE10846 [61,62] revealed a negative correlation between CD44 expression and overall survival (OS) probability in DLBCL patients, suggesting that an active MyD88^L265P^-NF-kB-CD44 axis might have novel prognostic and predictive value in DLBCL subsets. However, the functional consequences of the deregulated expression of CD44 in lymphomagenesis remain to be elucidated.

In summary, our study provides important insights into the molecular mechanisms of MyD88^L265P^ oncogenic signaling and their potential implications for lymphoma biology. Our working hypothesis is that the observed increased surface levels of CD44, as well as the deregulated expression of other genes from our RNA-seq dataset with reported involvement in biological processes related to cell adhesion and migration (such as LGALS3, CCL22, CD52, CROCC, and CTTLL3), might promote a more aggressive lymphoma cell phenotype and result in more disseminated malignancy. Future research is needed to understand the functional consequences of each of these MyD88^L265P^-deregulated genes in lymphomagenesis. Follow-up experimental work could include using gene engineering tools to overexpress or knock out these genes to investigate their specific roles in lymphoma cell migration in vitro and in vivo in mouse lymphoma models. Furthermore, it is tempting to speculate that the direct or indirect targeting of CD44 (and possibly other genes upregulated by MyD88^L265P^, such as LGALS3) could serve as potential novel targets for treatment that could improve the clinical outcome in MyD88^L265P^-driven malignancies. However, we emphasize that this is a hypothesis based on preclinical studies, and further research is needed to determine the clinical implications of our findings and the feasibility and efficacy of such potential interventions.

## 4. Materials and Methods

### 4.1. Cell Lines

Human cell lines U2932, HBL1, TMD8, OCI-Ly7, OCI-Ly18, and SUDHL6 were a kind gift from Dr. Ondrej Havranek (1st Faculty of Medicine of the Charles University, Prague, Czech Republic). The U2OS cell line was obtained from American Type Culture Collection (ATCC, Manassas, VA, USA; cat. No. HTB-96); THP1 Dual, from Invivogen (Invivogen, San Diego, CA, USA; cat. No. thpd-nfis); and HEK 293FT, from Invitrogen (cat. No. R70007). Cell lines were maintained in RPMI1640 medium (U2932, HBL1, TMD8, SUDHL6, and THP1 Dual), IMDM (OCI-Ly7 and OCI-Ly18), or DMEM (U2OS and 293FT) supplemented with 10% heat-inactivated fetal bovine serum (BioSera Europe, Cholet, France; cat. No. FB-1101/500) and 1% penicillin streptomycin (VWR, Radnor, PA, USA; cat. No. LONZ17-603E).

### 4.2. Cloning

The strategy for cloning MyD88 in the inducible vector was developed using SnapGene (version 4.3.11; SnapGene software; www.snapgene.com). As the backbone, we used pLVX-TetOne-Puro-hAXL [34], a gift from Kenneth Pienta (Addgene plasmid No. 124797; RRID:Addgene_124797). The hAXL sequence was excised using restriction endonucleases EcoRI-HF (NEB, Ipswich, MA, USA; cat. No. R3101S) and AgeI-HF (NEB; cat. No. R3552L). The digested products were dephosphorylated using FastAP Thermosensitive Alkaline Phosphatase (Thermo Scientific, Waltham, MA, USA; cat. No. EF0651) and extracted from 1% agarose gel in TAE buffer using a Gel extraction kit (Qiagen, Hilden, Germany; cat. No. 28706).

The MyD88 cDNA clone (BC013589; isoform 2 NM_002468.5 → NP_002459.3) was purchased from Dharmacon, Lafayette, CO, USA. The L265P mutation was introduced using oligonucleotide site-directed mutagenesis, using Phusion Site-Directed Mutagenesis Kit (Thermo Scientific, Waltham, MA, USA; cat. No. F541) and 5′-phosphorylated mutagenic specific primers (Supplementary Table S5) following the manufacturer’s instructions. The sequences were amplified with Q5 High-Fidelity 2× Master Mix (NEB, Ipswich, MA, USA; cat. No. M0492S) using a set of primers for introducing the restriction sites for EcoRI and AgeI, at 5′ and 3′, respectively (Supplementary Table S5). The amplified products were purified using a PCR purification kit (Qiagen, Hilden, Germany; cat. No. 28106) and then digested using EcoRI and AgeI restriction enzymes. The linearized vector and the digested PCR products (wt or L265P) were ligated using T4 DNA ligase (NEB, Ipswich, MA, USA; cat. No. M0202S). Subsequently, Stbl3 bacteria were transformed using the heat shock protocol. The transformed bacteria were plated on an agarose plate in the presence of ampicillin (100 μg/mL) and incubated overnight at 37 °C. The next day, the colonies were screened with PCR, using PPP Master Mix (Top-Bio, Vestec, Czech Republic; cat. No. P126) and a set of MyD88-specific primers (Supplementary Table S5). Positive clones were isolated using an E.Z.N.A. Endo-free plasmid mini II kit (VWR, Radnor, PA, USA; cat. No. D6950-02), and DNA was sequenced at Eurofins Genomics. All cloning steps were performed according to the manufacturers’ protocols.

### 4.3. Lentiviral Particle Production and Cell Transduction

Briefly, lentiviral constructs (1.64 pmol pLVX_TetOne) for the inducible overexpression of MyD88 (wt/L265P) were used for plasmid construction and transfected into 293FT cells (3 × 10^6^ cells seeded in a 10 cm dish overnight) together with helper plasmids (0.72 pmol pMD2.G and 1.3 pmol psPAX2) using PEI transfection reagent. Viral supernatants were collected 72 h post-transfection, filtered through a 0.45 µm syringe filter (VWR, Radnor, PA, USA; cat. No. 514-0063), mixed with PEG-8000 solution (final concentration of 10% *w*/*v*) and sodium chloride (final concentration of 0.3 M), and agitated overnight at 4 °C. The next day, viral particles were concentrated using centrifugation at 4000× *g* for 20 min (4 °C). The resulting pellet was resuspended in 200 µL of PBS and used for the infection of 2 × 10^6^ U2932, THP1 Dual, or U2OS cells. The cells were incubated with the viral supernatant in the presence of 10 μg/mL polybrene (Sigma-Aldrich, St. Louis, MO, USA; cat. No. TR-1003-G) in a final volume of 1 mL and spin-infected for 1 h at 900× *g* (34 °C). Cells were then supplemented with 9 mL of fresh medium, cultured for at least 48 h, and selected with puromycin (1 μg/mL) (Sigma-Aldrich, St. Louis, MO, USA; cat. No. P883-25MG) for 3 days.

### 4.4. Western Blotting

Whole-cell lysates were prepared in SDS lysis buffer (20 mM Tris-Cl (pH 7.5), 100 mM NaCl, and 5 mM EDTA (pH 8.0)) supplemented with Pierce universal nuclease (cat. No. 88701) and protease–phosphatase inhibitors (Thermo Scientific, Waltham, MA, USA; cat. No. A32961).

The lysates were resolved on SurePAGE Bis-Tris gradient gel at 4–12% (GenScript, Piscataway, NJ, USA; cat. No. M00653/M00654). The proteins were transferred to PVDF membranes. Membranes were blocked in 5% (*w*/*v*) non-fat milk (Carl Roth, Karlsruhe, Germany) in PBS-T (phosphate buffer saline with 0.05% Tween-20) and incubated overnight at 4 °C in 1% (*w*/*v*) BSA/PBS-T with the appropriate primary antibodies. The primary antibodies used at the indicated dilutions included anti-Actin C4 (Thermo Scientific, Waltham, MA, USA; cat. No. MA511869;), anti-p-NF-kappaB p65 (S536) (CST, Danvers, MA, USA; clone 93H1; cat. No. 3033S), anti-IkB-zeta (CST, Danvers, MA, USA; cat. No. 9244S), anti-IkB-alpha (CST, Danvers, MA, USA; cat. No. 9242S), anti-p-IkB-alpha (S32) (CST, Danvers, MA, USA; clone 14D4; cat. No. 2859S), anti-NF-kappaB p65 (CST, Danvers, MA, USA; clone D14E12; cat. No. 8242S), anti-MyD88 (CST, Danvers, MA, USA; clone D80F5; cat. No. 4283), anti-BATF (Santa Cruz Biotechnology, Dallas, TX, USA; cat. No. sc-100974X), and anti-CD44 (Abnova, Taipei, Taiwan; clone 156-3C11; cat. No. ABNOVAB12125).

Membranes were washed three times with PBS-T and incubated with HRP-conjugated secondary antibodies for 1 h at room temperature. The HRP-coupled secondary antibodies used at the indicated dilutions included goat anti-rabbit-IgG (111-035-144; Jackson ImmunoResearch, West Grove, PA, USA; 1:5000) and goat anti-mouse-IgG (115-035-146; Jackson ImmunoResearch; 1:5000). Then, the membranes were washed three times, and signal detection was performed using ECL (Thermo Scientific , Waltham, MA, USA) and ChemiDoc MP System (Bio-Rad, Hercules, CA, USA). Image Lab version 6.0.1 (BioRad), was used for the Western blot imaging elaboration and band intensity quantification.

### 4.5. THP1 Dual Reporter Assay

THP1 Dual cells were transduced with the pLVX-TetOne-GFP vector containing either MyD88 wt or L265P. The cells were plated in flat-bottom 96-well plates in triplicate at 1 × 10^5^ density in a final volume of 200 µL and induced for 24 h with DOX. Culture suspensions were collected, and the levels of the two secreted reporter proteins (SEAP (Secreted Embryonic Alkaline Phosphatase), for NF-kB activation, and Lucia luciferase, for IRF activation) were determined following the manufacturer’s instructions. Briefly, QUANTI-Blue Solution (Invivogen; cat. No. rep-qbs) can quantify SEAP activity, which is secreted by the cells in the culture medium. The enzyme-induced color change of the solution from pink to blue due to SEAP activity was detected by measuring the absorbance at 635 nm using Infinite F Plex (Tecan, Männedorf, Switzerland). QUANTI-Luc reagent (Invivogen; cat. No. rep-qlc2) was used to determine the levels of Lucia luciferase in the samples using a bioluminescent method. The light emitted upon reagent conversion was detected using Infinite F Plex (Tecan, Männedorf, Switzerland). GraphPad Prism, version 8.0.0 (GraphPad Software, San Diego, CA, USA; www.graphpad.com), was used for graph preparation.

### 4.6. RNA Isolation

Total RNA was extracted from cells using RNeasy Mini Kit (Qiagen, Hilden, Germany; cat. No. 74106). The RNA aliquots were stored at −80 °C. The RNA concentration was quantified using a Qubit 2.0 fluorometer (Life Technologies, ThermoFisher Scientific, Waltham, MA, USA), and the quality was assessed with Agilent 2200 Tapestation (Agilent Technologies, Santa Clara, CA, USA) using High Sensitivity RNA ScreenTape following the manufacturers’ instructions.

### 4.7. RNA Sequencing and Transcriptome Analysis

At least 2 µg of total RNA from each sample was sent to Macrogen Europe for TruSeq stranded mRNA library generation and RNA-seq analysis using the NovaSeq 6000 Illumina platform. The RNA-sequencing data were analyzed using an in-house Snakemake [154] pipeline. The raw fastq sequences were trimmed for adapter and low-quality reads using TrimGalore v0.6.6, a wrapper of the Cutadapt [155] program, and SortMeRNA v4.2.0 [156] was used for filtering out rRNA reads. Additionally, we tested sequencing data quality using STAR aligner v2.7.7a [157] followed by Qualimap v2.2.2-dev [158]. The reads that passed these quality control steps were then subjected to the quantification of transcripts using Salmon v1.4.0 [157]. The differentially expressed genes were summarized using the R package DESeq2 v1.30.0 [159]. DESeq2 results were visualized using R with the ggplot2 v3.3.3 [160] package. Significantly differentially expressed genes with Benjamini and Hochberg corrected *p*-value less than 0.05 and absolute value of log2 fold change greater than 0.5 were used for heatmap visualization.

### 4.8. Quantitative Real-Time Polymerase Chain Reaction

Complementary DNA (cDNA) synthesis was performed using RevertAid First Strand cDNA Synthesis Kit (Thermo Scientific, Waltham, MA, USA) according to the manufacturer’s instructions. Quantitative RT-PCR (qPCR) was conducted using PowerUp^TM^SYBR^TM^ Green Master Mix (Applied Biosystems, Waltham, MA, USA) with StrepOnePlus Real-Time PCR System (Applied Biosystems, Waltham, MA, USA). Relative mRNA expression was calculated using the 2^−ΔΔCt^ method and normalized to the HPRT gene. Oligonucleotide sequences used in the study can be found in Supplementary Table S6. GraphPad Prism, version 8.0.0 (GraphPad Software, San Diego, CA, USA; www.graphpad.com), was used for graph preparation.

### 4.9. Data Analysis for RNA-Seq Validation

For the validation of the expression of the top hits obtained in the RNA-seq analysis, we used (1) normalized expression derived from the gene expression profiling of an array of 7 lymphoma cell lines (GSE94669) [32] and (2) raw counts from RNA-seq data of 14 DLBCL samples (of which 7 samples had overexpressed mutated MYD88) (GSE141453) [147]. Raw counts from (2) were then normalized using Deseq2 for the final analysis.

### 4.10. CD44 Surface Phenotype

Cells were harvested, washed twice with PBS, and incubated at 4 °C in the dark for 25 min with the respective antibody. Two antibodies for CD44 were used for the different cell lines: FITC anti-mouse/human CD44 Antibody clone IM7 (Biolegend, San Diego, CA, USA; cat. No. 103006) and APC anti-mouse/human CD44 Antibody clone IM7 (Biolegend, San Diego, CA, USA; cat. No. 103011). After incubation, the cells were washed twice with PBS and then analyzed using flow cytometry with Cytoflex S (Beckman Coulter, IN, USA), and the data were acquired using CytExpert software v2.4 and analyzed using FlowJo v10 (FlowJo, OR, USA). GraphPad Prism, version 8.0.0 (GraphPad Software, San Diego, CA, USA; www.graphpad.com), was used for graph preparation.

### 4.11. Survival Analysis

The transcriptome profiling and clinical information of 449 DLBCL patients from GEO datasets (GSE10846; n = 420) [61,62] were used for overall survival (OS) analysis. The dataset contains 13 probes for CD44 expression. Therefore, for determining the indicative effect of the CD44 expression level on overall survival, the mean of all 13 probes for each patient was calculated. For this purpose, the cutoff was then calculated using the CutoffFinder algorithm to determine the optimal cutoff point for high and low expression of CD44. Specifically, the average expression matrix was uploaded to Cutoff Finder [161], and the cutoff value was determined using the “significance (Fisher’s exact test)” method. The cutoff values of high, expression > 9.726, and low, expression ≤ 9.726, were applied. The survival analyses (KM and Cox analyses) were performed using R packages survival v3.2.11, survminer v0.4.9, and tidyverse v1.3.1. The OS analysis of every single probe was obtained from http://www.genomicscape.com/, accessed on the 11 November 2022.

### 4.12. Statistical Analysis

The statistical significance of differences among various groups was calculated using the two-tailed paired *t*-test, and error bars represent the standard deviation (SD). Statistical analyses, unless otherwise indicated, were performed using GraphPad Prism 8. Data are shown as means ± SD. Images of gels in the figures show representative experiments that were repeated as independent biological replicates a minimum of three times.

## 5. Conclusions

In conclusion, we investigated the transcriptional response to inducible MyD88^L265P^ oncogenic signaling in the model U2932 lymphoma cell line. Our RNA-seq analysis identified NF-κB-regulated genes that might contribute to lymphomagenesis, including CD44, LGALS3 (coding Galectin-3), NFKBIZ (coding IkBƺ), and BATF. Moreover, we demonstrated that CD44 could serve as a marker of ABC-DLBCL and that CD44 expression is correlated with overall survival in DLBCL patients. Thus, our analysis provides new insights into the downstream outcomes of MyD88^L265P^ oncogenic signaling, which might be involved in cellular transformation and provide novel therapeutical targets.

## Data Availability

The original contributions presented in the study are included in the article and Supplementary Material. Further inquiries can be directed to the corresponding author.

## Supplementary Materials

The following supporting information can be downloaded at: https://www.mdpi.com/article/10.3390/ijms24065623/s1. References [162,163,164,165,166,167,168,169,170,171,172,173,174,175,176,177,178,179,180,181,182,183,184,185,186,187,188,189,190,191,192,193,194,195,196,197,198,199,200,201,202] are cited in the supplementary materials.
