# Supplementary material for: Transcriptome Analysis of Diffuse Large B-Cell Lymphoma Cells Inducibly Expressing MyD88 L265P Mutation Identifies Upregulated CD44, LGALS3, NFKBIZ, and BATF as Downstream Targets of Oncogenic NF-κB Signaling
Source: Int J Mol Sci. 2023 Mar 15;24(6):5623. doi: 10.3390/ijms24065623 (PMC10057398; doi:10.3390/ijms24065623)
Supplement: Supplementary file 1 [file ijms-24-05623-s001.zip › ijms-2244850-Figures S1-S6.pdf]

Figure S1

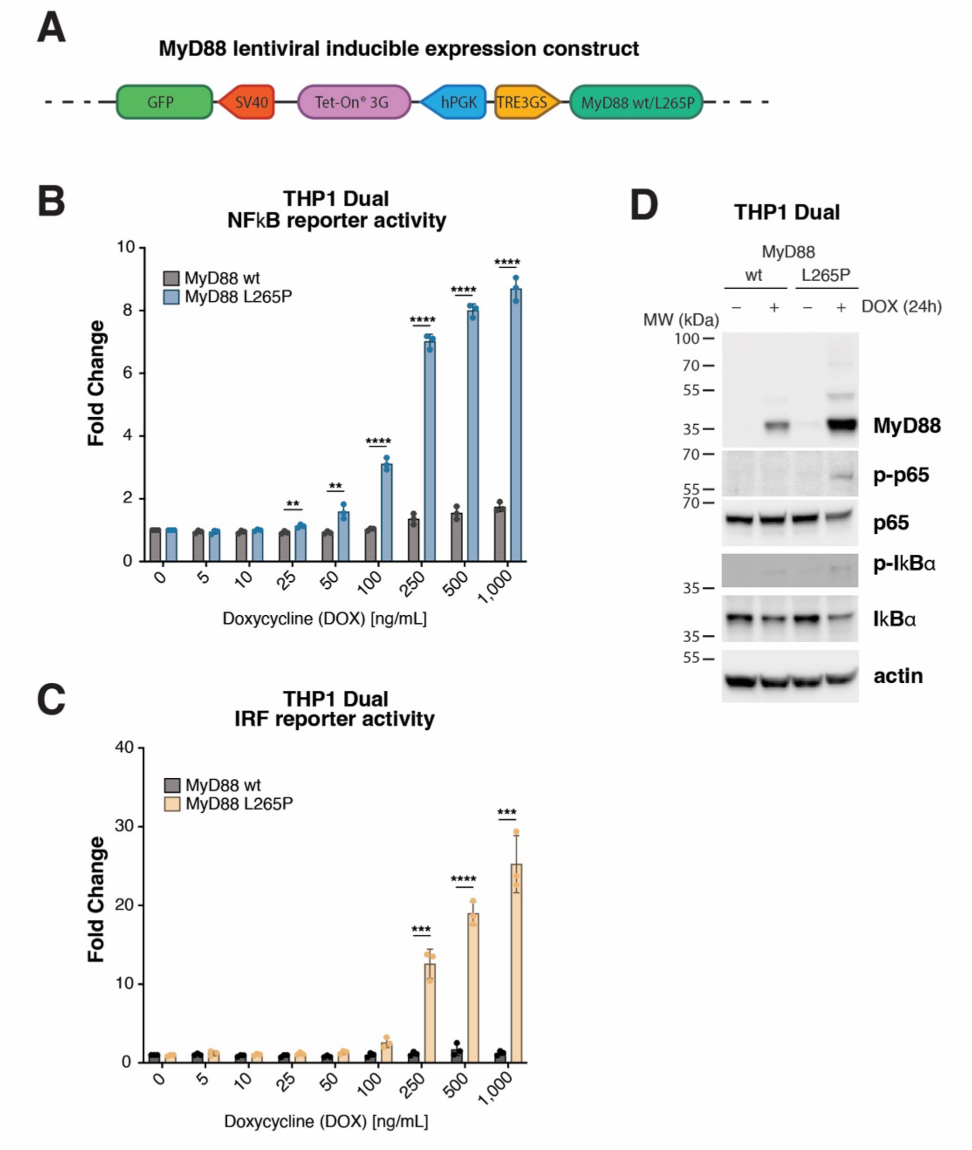

Supplementary Figure S1. **Development of an inducible system to express MyD88 wt or L265P in THP1-Dual cell line. (A)** Schematic representation of the tetracycline-inducible lentiviral gene expression system, pLVX-Tet-One-GFP, used for the overexpression of MyD88 (wt/L265P) in stable cell lines. **(B)** Doxycycline dose titration for the NF-kB activation in THP1 Dual cell line. NF-kB activation was measured by adding QUANTI-Blue solution to medium conditions, and absorbance at 635 nm was measured for each sample. **(C)** Doxycycline dose titration for the IRF activation in THP1 Dual cell line. IRF activation was measured by adding QUANTI-Luc solution to medium conditions, and luminescence was measured for each sample. **(D)** Western blot analysis of MyD88 inducible expression after 24 h of DOX [250 ng/ml] treatment in THP1 Dual cell line. Blotting for p-p65, p65, p-

Ikα and IkBα was used to determine NF-kB pathway activation. Actin was used as a loading control.

(** for $p \leq 0.01$, *** for $p \leq 0.001$, **** for $p \leq 0.0001$)

Figure S2

A

U2932 cells - RNA-seq differentially expressed genes norm. counts

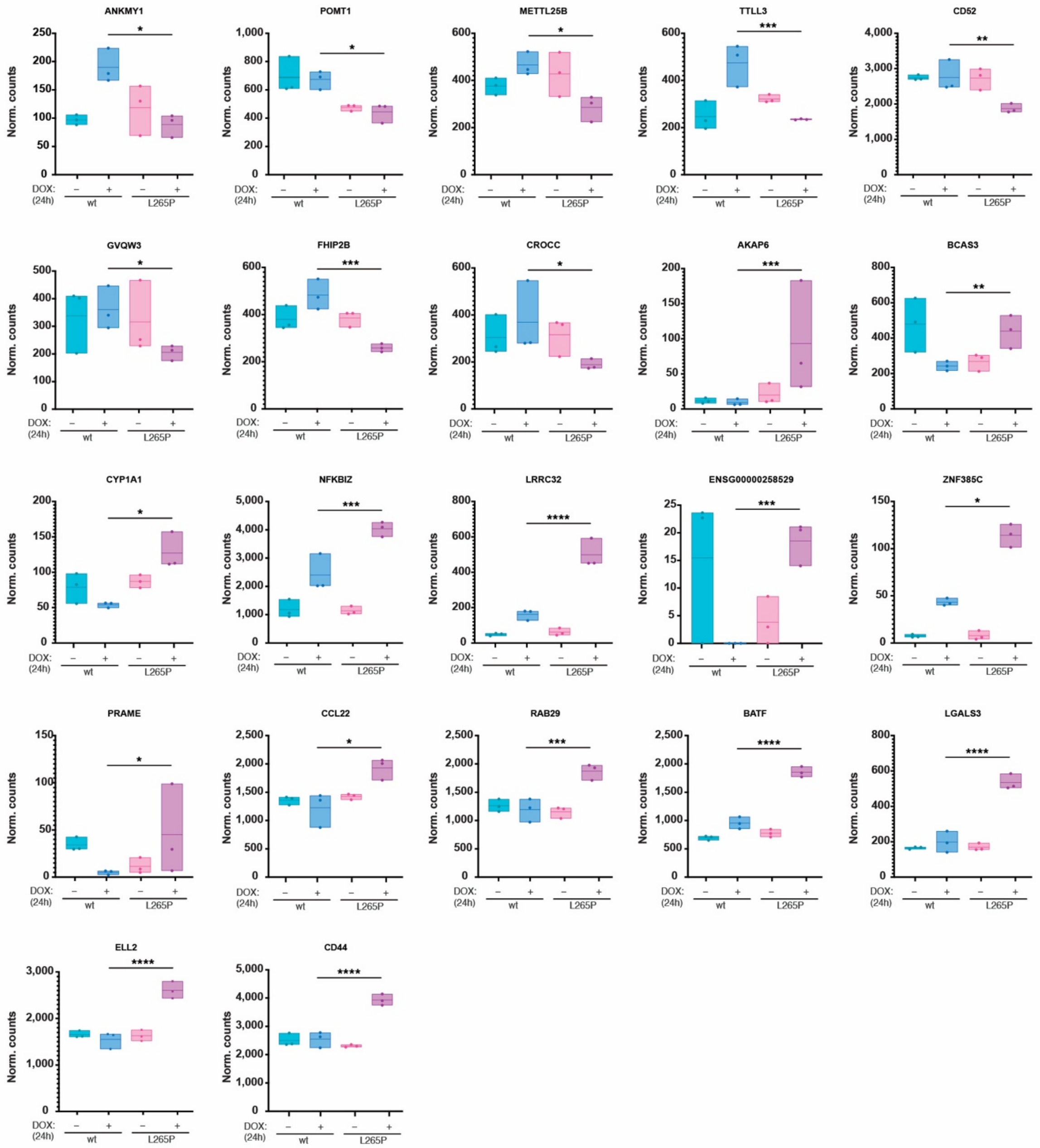

Figure S2

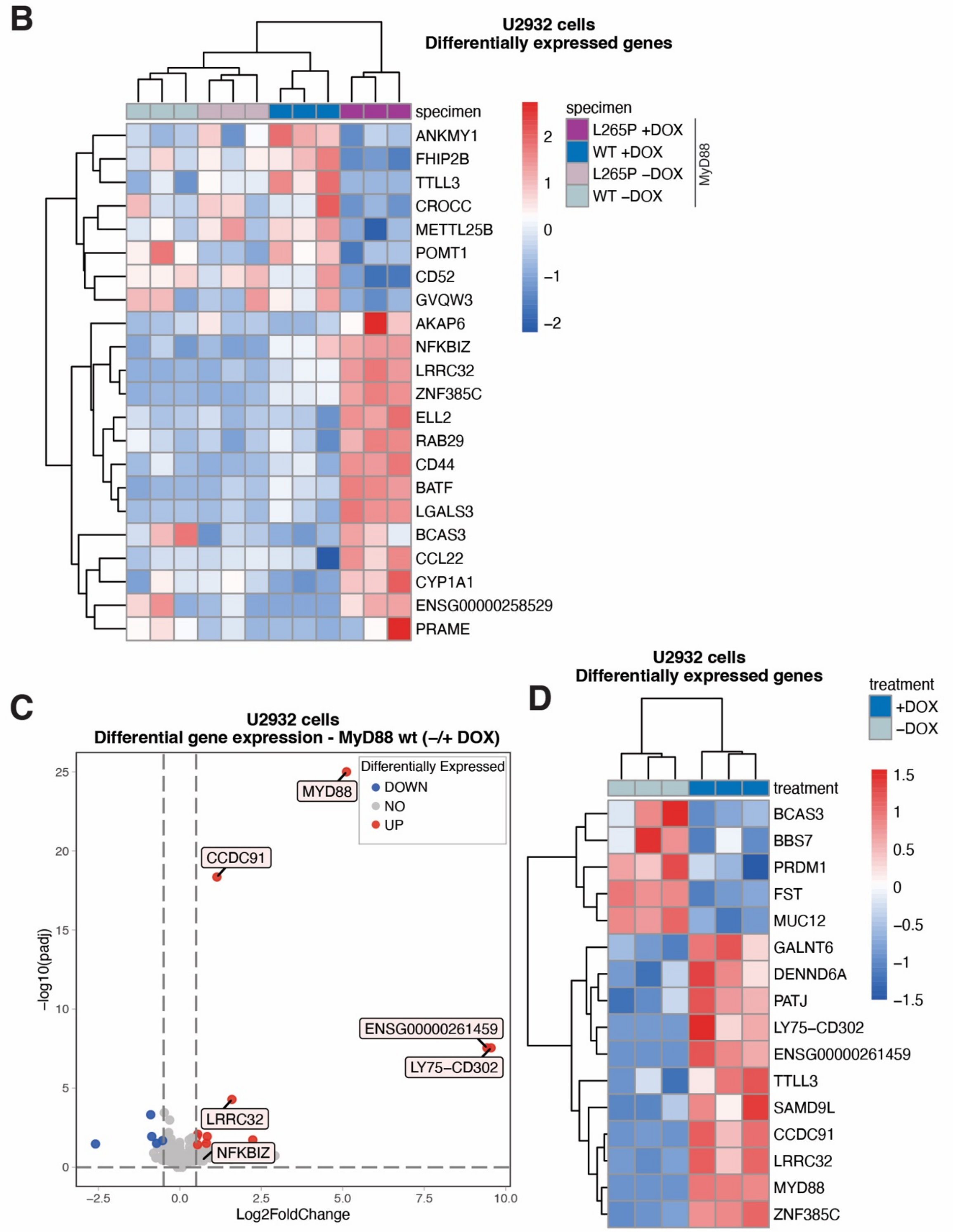

B
U2932 cells
Differentially expressed genes
specimen
ANKMY1
FHIP2B
TTLL3
CROCC
METTL25B
POMT1
CD52
GVQW3
AKAP6
NFKBIZ
LRRC32
ZNF385C
ELL2
RAB29
CD44
BATF
LGALS3
BCAS3
CCL22
CYP1A1
ENSG00000258529
PRAME
2
1
0
−1
−2
specimen
L265P +DOX
WT +DOX
L265P −DOX
WT −DOX
MyD88
C
U2932 cells
Differential gene expression - MyD88 wt (−/+ DOX)
Differentially Expressed
DOWN
NO
UP
MYD88
CCDC91
ENSG00000261459
LY75−CD302
LRRC32
NFKBIZ
−log10(padj)
Log2FoldChange
25
20
15
10
5
0
−2.5
0.0
2.5
5.0
7.5
10.0
D
U2932 cells
Differentially expressed genes
treatment
+DOX
−DOX
treatment
BCAS3
BBS7
PRDM1
FST
MUC12
GALNT6
DENND6A
PATJ
LY75−CD302
ENSG00000261459
TTLL3
SAMD9L
CCDC91
LRRC32
MYD88
ZNF385C
1.5
1
0.5
0
−0.5
−1
−1.5

Figure S2

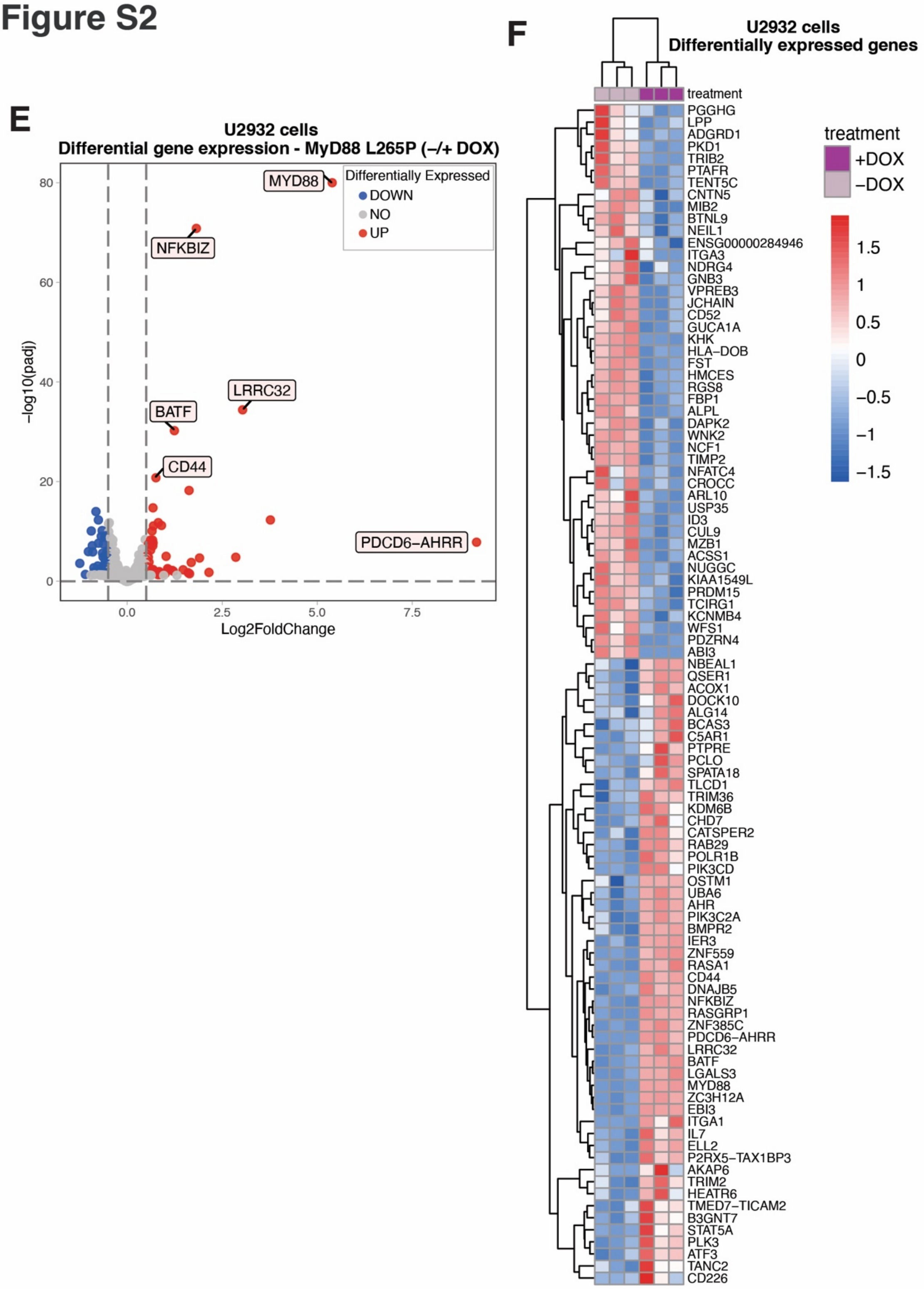

Supplementary Figure S2. Transcriptome analysis (RNA-seq) of U2932 lymphoma cell lines expressing MyD88 wt or MyD88^{L265P}. (A) Normalized counts from the transcriptome analysis for all the 22 most significantly deregulated genes showed in Figure 3B. **(B)** Heatmap representation of all

significantly differentially expressed genes (with the same thresholds as in volcano plot) in U2932 cell lines induced wt vs. induced MyD88 L265P, for all the four groups analysis (MyD88 wt untreated, wt induced, L265P untreated and L265P induced). **(C)** Volcano plot with differentially expressed genes in U2932 cell lines untreated (-DOX) vs. inducible expressing MyD88 wt (+DOX). The blue dots indicate downregulated genes, and the red dots indicate upregulated genes, with Benjamini and Hochberg corrected p value less than 0.05 and an absolute log2 foldchange greater than 0.5. **(D)** Heatmap representation of all significantly differentially expressed genes (with the same thresholds as in volcano plot) in U2932 cell lines untreated vs. inducible expressing MyD88 wt. **(E)** Volcano plot with differentially expressed genes in U2932 cell lines untreated vs. inducible expressing $MyD88^{L265P}$. The blue dots indicate downregulated genes, and the red dots indicate upregulated genes, with Benjamini and Hochberg corrected p value less than 0.05 and an absolute log2 fold-change greater than 0.5. **(F)** Heatmap representation of all significantly differentially expressed genes (with the same thresholds as in the volcano plot) in U2932 cell lines untreated vs. inducible expressing $MyD88^{L265P}$. Note: In (C) and (E), MyD88 had an adjusted p value 0, so we artificially put MyD88 at the top of the graph to visualize the fold change.

(* for $p \leq 0.05$, ** for $p \leq 0.01$, *** for $p \leq 0.001$, **** for $p \leq 0.0001$)

Figure S3

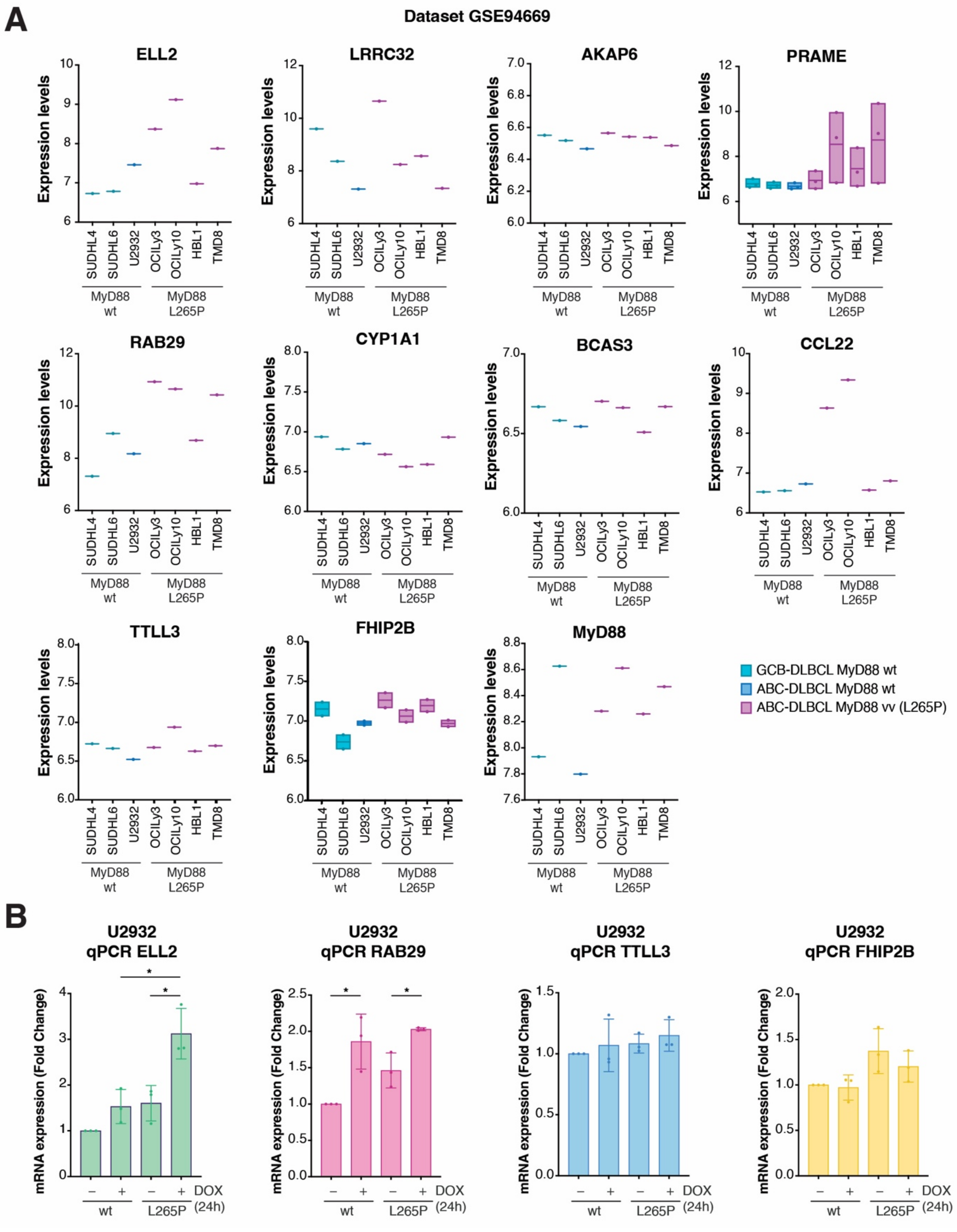

Supplementary Figure S3. Validation of transcriptome analysis results using publicly available datasets and in the model cell lines. (A) Gene expression validation in the immortalized cell lines, using the data from the dataset GSE94669 for the upregulated genes not shown in Figure 3, and the

two most significantly downregulated genes, in the comparison to U2932 inducibly expressing MyD88 wt vs. $MyD88^{L265P}$). As described in Figure 2, from the whole dataset containing the gene expression of 61 cell lines, we used only 7 representative cell lines. (**B**) qPCR validation of differentially expressed genes *ELL2*, *RAB29*, *TTLL3,* and *FHIP2B* in U2932 cell line upon MyD88 (wt/L265P) inducible expression after 24 h of DOX treatment [250 ng/mL] (* for $p \leq 0.05$).

Figure S4

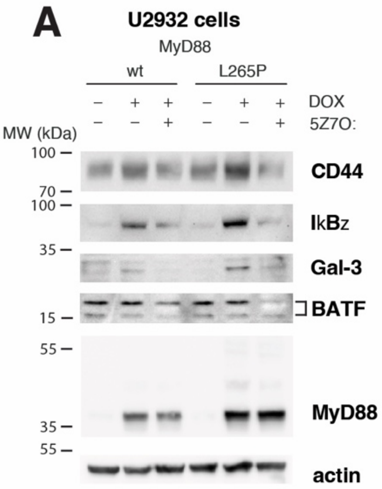

Supplementary Figure S4. Validation of the effect of 5Z7O [1 μM] at the protein level in U2932 cell lines. (A) Western blotting of MyD88 expression levels in U2932 cell lines after 24 h DOX [250 ng/mL] and subsequent 24 h 5Z7O [1 μM] treatment for the protein levels of BATF, LGALS3, NFKBIZ, and CD44.

Figure S5

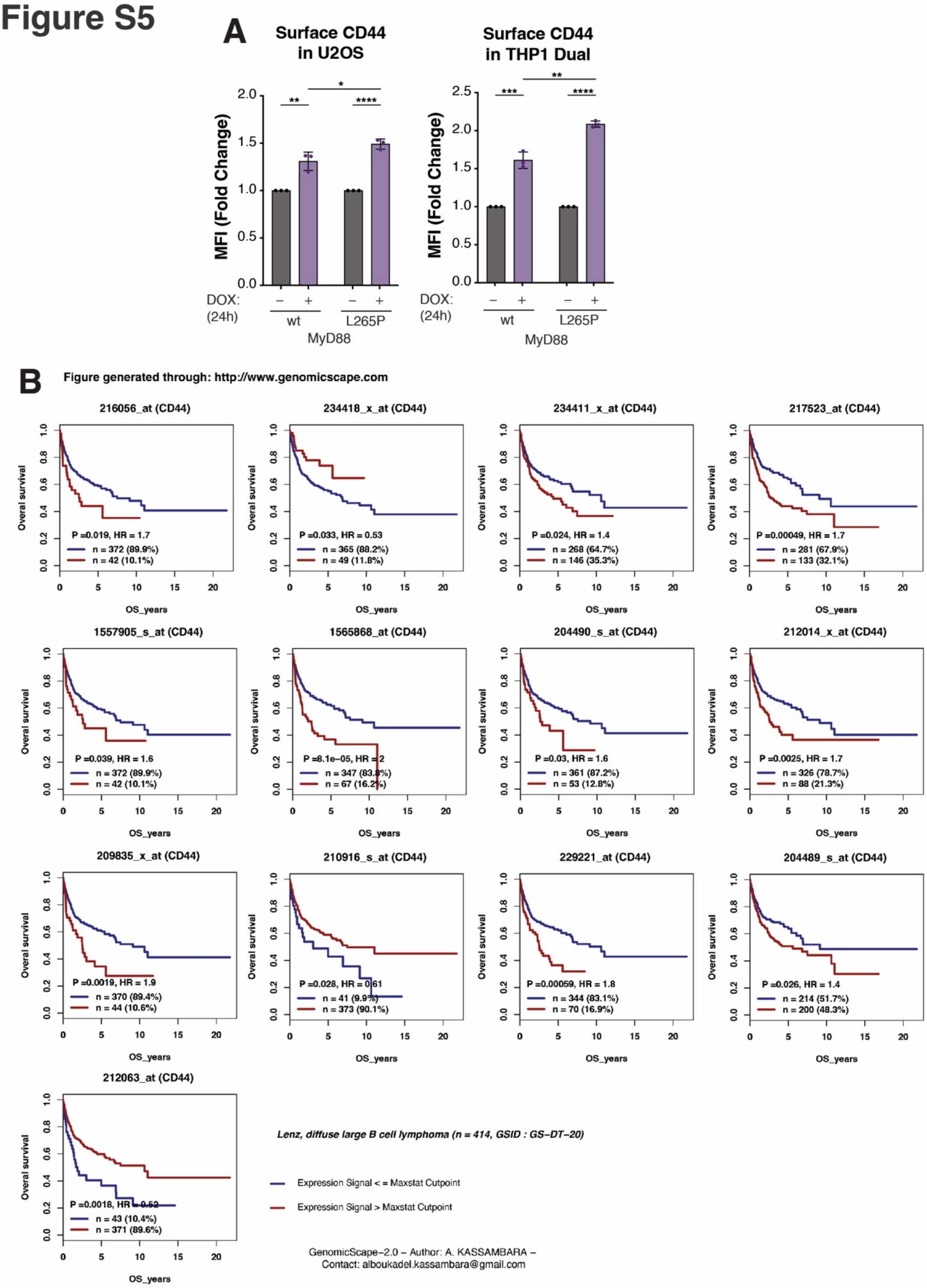

Supplementary Figure S5. Surface staining for CD44 and CD44-related OS in DLBCL patients from Lenz dataset GSE10846. (A) MFI for FITC obtained from the flow cytometry analysis of U2OS and THP1 Dual cell lines, upon MyD88 (wt/L265P) inducible expression, after 24 h of DOX treatment

[250 ng/mL], stained with CD44-FITC conjugated antibodies (* for $p \leq 0.05$, ** for $p \leq 0.01$, *** for $p \leq 0.001$, **** for $p \leq 0.0001$). **(B)** OS of DLBCL patients using the transcriptome profiling and clinical information of 449 DLBCL patients from GEO datasets (GSE10846, n = 420). The OS for each probe available in the dataset were generated through website www.genomicscape.com.

Figure S6

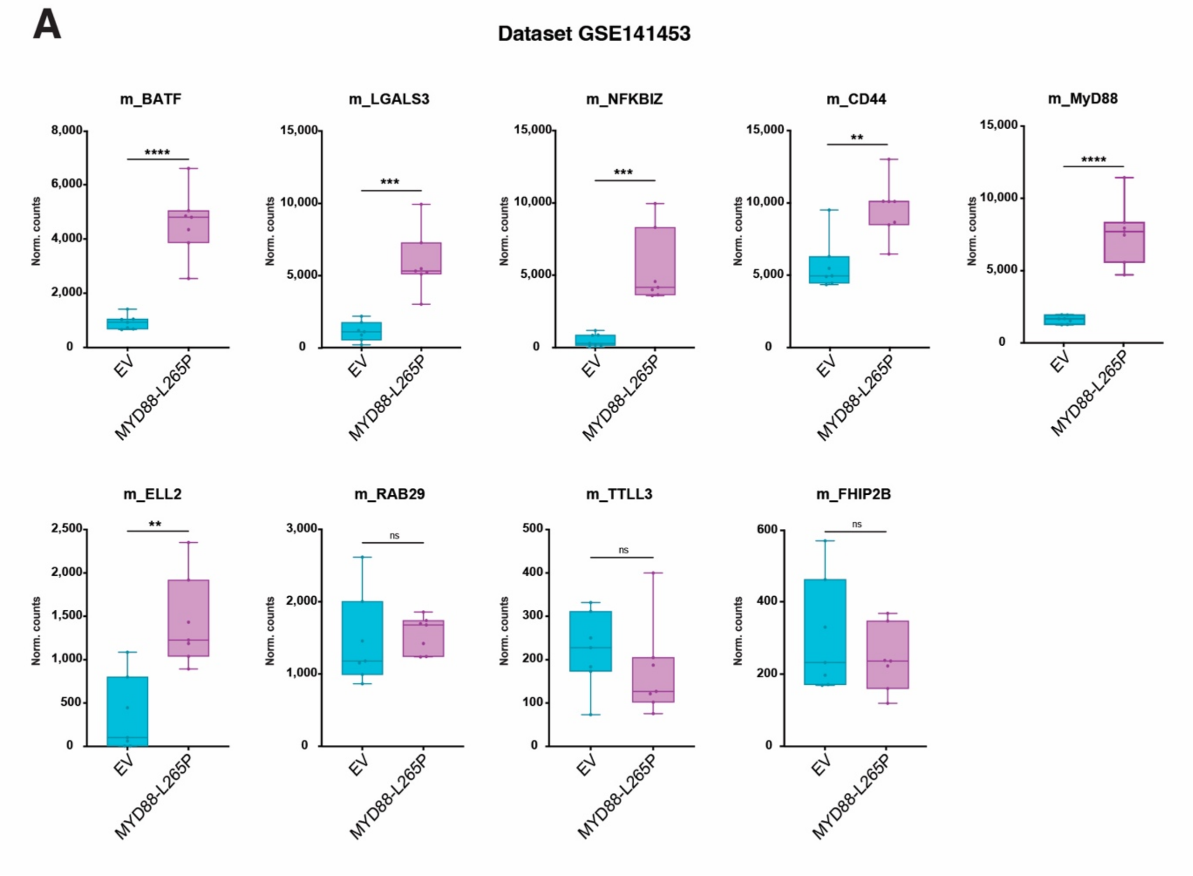

Supplementary Figure S6. Validation of transcriptome analysis results using publicly available datasets in a murine model. (A) Gene expression validation in mouse lymphoma model with MyD88^{L265P} expression compared to empty vector control (EV), using the data from the dataset GSE141453 for the genes BATF, LGALS3, NFKBIZ, CD44, *ELL2, RAB29* (upregulated in the comparison of U2932 inducible expressing MyD88 wt vs. MyD88^{L265P}), and *TTLL3* and *FHIP2B* (downregulated in the comparison of U2932 inducible expressing MyD88 wt vs. MyD88^{L265P}) and MyD88 (ns for $p > 0.05$, ** for $p \leq 0.01$, *** for $p \leq 0.001$, **** for $p \leq 0.0001$).
