# Supplementary material for: Transcriptome Analysis of Diffuse Large B-Cell Lymphoma Cells Inducibly Expressing MyD88 L265P Mutation Identifies Upregulated CD44, LGALS3, NFKBIZ, and BATF as Downstream Targets of Oncogenic NF-κB Signaling
Source: Int J Mol Sci. 2023 Mar 15;24(6):5623. doi: 10.3390/ijms24065623 (PMC10057398; doi:10.3390/ijms24065623)
Supplement: Supplementary file 1 [file ijms-24-05623-s001.zip › Table S3 Univariate and multivariate analysis for CD44 in the dataset GSE10846.pdf]

**Supplementary Table S3**. Univariate and multivariate analysis for CD44 in the dataset GSE10846

| | Univariate analysis | | | Multivariate analysis | | |
|---|---|---|---|---|---|---|
| | N | HR (95% CI for HR) | p.value | N | HR (95% CI for HR) | p.value |
| **Age** | | | | | | |
| <= median(62.5) | 207 | - | - | 207 | - | - |
| > median(62.5) | 207 | 2.2 (1.6-3.1) | <0.001 | 207 | 2.2 (1.6-3.1) | <0.001 |
| **Sex** | | | | | | |
| Female | 172 | - | - | 172 | - | - |
| Male | 224 | 1 (0.74-1.4) | 0,9 | 224 | 1.2 (0.8-1.6) | 0,3 |
| **Treatment** | | | | | | |
| CHOP-Like Regimen | 181 | - | - | 181 | - | - |
| R-CHOP-Like Regimen | 233 | 0.52 (0.38-0.72) | <0.001 | 233 | 0.57 (0.4-0.8) | <0.001 |
| **CD44 expression** | | | | | | |
| Low | 295 | - | - | 295 | - | - |
| High | 125 | 1.6 (1.2-2.2) | 0,0029 | 125 | 1.6 (1.2-2.2) | 0,004 |
