# Supplementary material for: Transcriptome Analysis of Diffuse Large B-Cell Lymphoma Cells Inducibly Expressing MyD88 L265P Mutation Identifies Upregulated CD44, LGALS3, NFKBIZ, and BATF as Downstream Targets of Oncogenic NF-κB Signaling
Source: Int J Mol Sci. 2023 Mar 15;24(6):5623. doi: 10.3390/ijms24065623 (PMC10057398; doi:10.3390/ijms24065623)
Supplement: Supplementary file 1 [file ijms-24-05623-s001.zip › Table S4 Information of 22 differentially expressed genes .pdf]

Supplementary Table S4. Information of 22 differentially expressed genes in U2932 cell lines inducible expressing MyD88 vs. MyD88^{L265P}.

	log2 FoldChange	Gene symbol	Gene name	Alternative protein names	Molecular function	Reported in cancer and/or human pathology	Reported in Lymphoma
Downregulated by MyD88-L265P expression	-0,9211331	ANKMY1	Ankyrin Repeat And MYND Domain Containing 1	ZMYND13<br>Zinc Finger MYND Domain-Containing Protein 13<br>TSAL1<br>Testis-Specific Ankyrin-Like Protein 1<br>FLJ20499	unknown	Biliary atresia [162]<br><br>Low expression associated with shorter metastasis-free survival time in Osteosarcoma [163]	No
	-0,59831331	METTL25B	Methyltransferase Like 25B	C1orf66<br>CGI-41<br>RRNAD1<br>Ribosomal RNA Adenine Dimethylase Domain-Containing Protein 1<br>Ribosomal RNA Adenine Dimethylase Domain Containing 1	unknown		No
	-0,68905052	GVQW3	GVQW Motif Containing 3		unknown		No

	-0,83377977	FHIP2B	FHF Complex Subunit HOOK Interacting Protein 2B	FAM160B2<br>RAI16<br>Family With Sequence Similarity 160 Member B2<br>Retinoic Acid Induced Protein 16<br>FLJ21801	unknown	Can enhance the tumor-promoting effect [164]	No
	-0,50564632	POMT1	Protein O-Mannosyltransferase 1	LGMD2K<br>Dolichyl-Phosphate-Mannose--Protein Mannosyltransferase 1<br>EC 2.4.1.109<br>LGMDR11<br>MDDGA1<br>MDDGB1<br>MDDGC1<br>RT	Synthesizing of O-mannosyl [165]	Walker-Warburg syndrome [166]<br><br>Leukemia [168]<br><br>Acute myeloid leukemia and Kidney tumor [168]	No
	-0,94536525	TTLL3	Tubulin Tyrosine Ligase Like 3	HOTTL<br>Tubulin Monoglycylase TTLL3<br>DKFZP434B103<br>EC 6.3.2.-	Regulates the stability and function of microtubules [169]	Ovarian cancer [170]	No

	-0,81900454	CROCC	Ciliary Rootlet Coiled-Coil	Rootlein<br>TAX1BP2<br>ROLT<br>KIAA0445<br>Tax1-Binding Protein 2<br>CROCC1	Formation and maintenance of cilia<br>cell movement and the sensing of the environment [171]<br><br>Centriole linker [172]<br><br>Centrosome cohesion [173]	Dysfunction of cilia has been linked to the development of certain types of cancer, including ovarian, pancreatic, and breast cancer [174–176], and CROCC may play a role in this process	No
	-0,50317365	CD52	CD52	HE5<br>EDDM5<br>CDW52<br>Human Epididymis-Specific Protein 5<br>Epididymal Secretory Protein E5<br>Cambridge Pathology 1 Antigen<br>CAMPATH-1 Antigen<br>CDW52 Antigen<br>HEL-S-171mP<br>CDw52	Regulate immune responses [177]	Lymphoma and leukemia [70]<br><br>Chronic lymphocytic leukemia (CLL) therapy [69]	Yes

				Epididymis Secretory Sperm Binding Protein Li 171mP			
Upregulated by MyD88-L265P	7,40836139	ENSG00000258529	Novel Protein	Mannosyltransferase<br>EC 2.4.1.-<br>PUS10	unknown	-	No
	1,234046639	ZNF385C	Zinc Finger Protein 385C	CTD-2132N18.2<br>CTD-2132N18.4	Nucleic acid binding activity and zinc ion binding activity.<br><br>Regulates p53 functions in cell cycle arrest [178]	-	No

	2,718040483	PRAME	Preferentially Expressed Antigen In Melanoma	CT130<br>MAPE<br>Melanoma Antigen Preferentially Expressed In Tumors<br>PRAME Nuclear Receptor Transcriptional Regulator<br>Preferentially Expressed Antigen Of Melanoma<br>Cancer/Testis Antigen 130<br>Opa-Interacting Protein 4<br>OIP4<br>Opa-Interacting Protein OIP4	Substrate-recognition component of a Cul2-RING (CRL2) E3 ubiquitin-protein ligase complex and regulation of NFY promoters [179]<br><br>Transcriptional repressor, inhibiting the signaling of retinoic acid through the retinoic acid receptors RARA, RARB and RARG [180]	Regulates cytotoxic drug sensitivity in Hodgkin Lymphoma [74]<br><br>Acute myelogenous leukemia (AML) and multiple myeloma (MM) and other hematological malignancies [72]<br><br>Leukemia [73]<br><br>In DLBCL interact with EZH2 protein and its deletions were associated with poor outcomes [79]	Yes

	0,715520862	ELL2	Elongation Factor For RNA Polymerase II 2	MRCCAT1 Metastatic Renal Cell Carcinoma-Associated Transcript 1 RNA Polymerase II Elongation Factor ELL2 Elongation Factor, RNA Polymerase II, 2 ELL-Related RNA Polymerase II, Elongation Factor Elongation Factor RNA For Polymerase II 2	Elongation factor for RNA polymerase II [82]<br><br>Involved in antibody secretion [80]<br><br>Unfolded protein response [81]	Prostate ([181,182]<br><br>Multiple myeloma [183,184]<br><br>In ABC DLBCL, EEL2 represents one of the enrichment signature genes [83]	Yes

	1,05058413	CYP1A1	Cytochrome P450 Family 1 Subfamily A Member 1	P450DX<br>P1-450<br>P450-C<br>CP11<br>Cytochrome P450, Subfamily I (Aromatic Compound-Inducible), Polypeptide 1<br>Hydroperoxy Icosatetraenoate Dehydratase<br>Cytochrome P450 Form 6<br>Cytochrome P450 1A1<br>Cytochrome P450-P1<br>Cytochrome P450-C<br>EC 1.14.14.1<br>CYPIA1<br>CYP1<br>Cytochrome P1-450, Dioxin-Inducible<br>Flavoprotein-Linked Monooxygenase<br>Aryl Hydrocarbon	Metabolism of certain drugs, detoxification of harmful substances, and activation of polycyclic aromatic hydrocarbons (PAHs) found in tobacco smoke and other environmental pollutants [185]	Polymorphisms in CYP1A1 might associate with increased DLBCL risk [186]	Yes

				Hydroxylase Xenobiotic Monooxygenase EC 4.2.1.152 AHRR AHH			
	0,598514207	RAB29	RAB29, Member RAS Oncogene Family	RAB7L1 RAB7, Member RAS Oncogene Family-Like 1 Ras-Related Protein Rab-7L1 Ras-Related	Vesicular trafficking regulates T cell receptor recycling	Parkinson’s Disease [188]	No

				Protein Rab-29 Rab-7-Like Protein 1 RAB7L	primary cilium assembly [187]		
	2,977058501	AKAP6	A-Kinase Anchoring Protein 6	AKAP100 MAKAP PRKA6 KIAA0311 ADAP6 ADAP100	Regulation of cellular signaling pathways [189,190]	Cardiovascular disease [191]	No
	0,757418431	BCAS3	Breast Carcinoma-Amplified Sequence 3	BCAS3 Microtubule Associated Cell Migration Factor PHAF2 Phagophore Assembly Factor 2 FLJ20128 Rudhira GAOB1 Breast Carcinoma Amplified Sequence 4/3 Fusion Protein Metastasis Associated Antigen Of Breast Cancer	Regulator of cell growth, apoptosis, and chemoresistance as a part CRL4A complex mediating ubiquitination and proteasomal degradation of p53 [192]	Breast cancer with poor prognosis [192,193]	No

				Breast Carcinoma Amplified Sequence 3 HEMARS MAAB			
	1,580603439	LRRC32	Leucine Rich Repeat Containing 32	GARP Glycoprotein A Repetitions Predominant D11S833E Transforming Growth Factor Beta Activator LRRC32 Leucine-Rich Repeat-Containing Protein 32 Garpin CPPRDD	Involved in immunosuppressive cytokine TGF-β in immune cells, including T regs, platelets [84,85,194]	Immunosuppressive tumor microenvironment [195]<br><br>Role on regulatory T cells in lung cancer [196]	No

	0,576702076	CCL22	C-C Motif Chemokine Ligand 22	MDC<br>A-152E5.1<br>DC/B-CK<br>STCP-1<br>ABCD-1<br>SCYA22<br>Small Inducible Cytokine Subfamily A (Cys-Cys), Member 22<br>CC Chemokine STCP-1<br>MGC34554<br>Stimulated T Cell Chemotactic Protein 1<br>Stimulated T-Cell Chemotactic Protein 1	Immunoregulatory and inflammatory processes [197]<br><br>Ligand for CCR4 receptor [93]	Head and neck squamous cell carcinoma [198,199]<br><br>Breast cancer [197,200]<br><br>Lung cancer [201]<br><br>Colorectal adenocarcinoms [202]<br><br>Hodgkin lymphoma [92,94]<br><br>T-cell Lymphoma [93]<br><br>B-cell lymphoproliferative disorder [95]<br><br>In DLBCL, CCL22 has been described in the gene enrichment signature [83,96]	Yes

	0,932599387	BATF	Basic Leucine Zipper ATF-Like Transcription Factor	B-ATF<br>SFA-2<br>BATF1<br>Basic Leucine Zipper Transcription Factor, ATF-Like<br>B-Cell-Activating Transcription Factor<br>SFA2<br>SF-HT-Activated Gene 2 Protein	Transcriptional regulators modulation of AP-1 transcription complex [97]	Anaplastic large cell lymphoma [101]<br><br>High BATF expression was demonstrated in DLBCL samples [106] and is considered a part of the gene enrichment signature of ABC DLBCL [83]	Yes

	0,696590349	NFKBIZ	NFKB Inhibitor Zeta	MAIL<br>INAP<br>IL-1 Inducible Nuclear Ankyrin-Repeat Protein B-Cells Inhibitor, Zeta<br>I-Kappa-B-Zeta<br>IkappaBzeta<br>FLJ34463<br>IKBZ<br>IkB-Zeta	Regulator of inflammation, cell proliferation and survival [110]	Bladder cancer [113]<br><br>High IκBζ expression was explicitly detected in ABC DLBCL [45]. Moreover, amplification of the NFKBIZ locus has been observed in ~10% of ABC DLBCL cases [114]<br><br>Overexpression of the IκBζ protein, which activates the NF-κB signaling pathway and provides a selective advantage to tumor cells [115,116]<br><br>The increase of NFKBIZ transcript and IκBζ protein was demonstrated due to constitutive oncogenic NF-κB signaling in MyD88L265P or CARD11L244P-expressing lymphoma cells [45]	Yes

	1,370455524	LGALS3	Galectin 3	GALIG Advanced Glycation End-Product Receptor 3 Lectin, Galactoside-Binding, Soluble, 3 Carbohydrate-Binding Protein 35 Galactose-Specific Lectin 3 Laminin-Binding Protein IgE-Binding Protein Lectin L-29 LGALS2 GALBP MAC2 Epididymis Secretory Sperm Binding Protein Galactoside-Binding Protein Mac-2 Antigen CBP 35 CBP35 Gal-3 GAL3 L-31	Cell adhesion, proliferation, differentiation, apoptosis, signaling, and immune system function [119,120]	Acute myeloid leukemia (AML) [124]<br><br>Chronic lymphocytic leukemia (CLL) [125]<br><br>Prognostic factor in Hodgkin's lymphoma [126]<br><br>In DLBCL, multiple studies associated overexpressed Gal-3 with increased cell proliferation, survival, and disease aggressiveness and identified GAL-3 as a prognostic factor and potential target for therapy [127,130–132]	Yes

	0,594912298	CD44	CD44 Molecule (Indian Blood Group)	HUTCH-I<br>HCELL<br>CSPG8<br>MC56<br>Pgp1<br>MDU2<br>MDU3<br>MIC4<br>IN<br>Hematopoietic Cell E- And L-Selectin Ligand<br>GP90 Lymphocyte Homing/Adhesion Receptor<br>Chondroitin Sulfate Proteoglycan 8<br>Extracellular Matrix Receptor III<br>Homing Cell Adhesion Molecule<br>Heparan Sulfate Proteoglycan<br>Phagocyte Glycoprotein 1<br>Hyaluronate Receptor<br>In(Lu) Related-P80<br>Hermes Antigen<br>Hermes-1	Multifunctional transmembrane receptor binding to various ligands, including hyaluronic acid (HA), collagen, and osteopontin, which can modulate its activity. Cell adhesion and migration, and it is involved in the activation and regulation of the immune system and the formation and maintenance of the extracellular matrix [139,142]<br><br>Cell dissemination [143]	Hodgkin andnon-Hodgkin lymphoma [59]<br><br>Prognostic factor in non-Hodgkin's lymphoma [55]<br><br>In ABC DLBCL, CD44 was identified as a part of the gene enrichment signature [83]	Yes

				ECMR-III<br>HUTCH-1<br>ECM-III<br>Epican<br>PGP-1<br>CD44R<br>CDw44<br>H-CAM<br>LHR<br>Extracellular Matrix Receptor-III<br>Indian Blood Group Antigen<br>Phagocytic Glycoprotein I<br>CDW44<br>PGP-I				

Reference in the main text
