# Supplementary material for: Transcriptome Analysis of Diffuse Large B-Cell Lymphoma Cells Inducibly Expressing MyD88 L265P Mutation Identifies Upregulated CD44, LGALS3, NFKBIZ, and BATF as Downstream Targets of Oncogenic NF-κB Signaling
Source: Int J Mol Sci. 2023 Mar 15;24(6):5623. doi: 10.3390/ijms24065623 (PMC10057398; doi:10.3390/ijms24065623)
Supplement: Supplementary file 1 [file ijms-24-05623-s001.zip › Table S5 List of cloning primers.pdf]

Supplementary Table S5. List of cloning primers.

Primer name	Sequence (5' -> 3')
MyD88 mutagenesis Fwd	GTACTTGATGGGGATCGGTCGCTTCTGATGGGC
MyD88 mutagenesis Rev	GCCCATCAGAAGCGACCGATCCCCATCAAGTAC
MyD88 EcoRI Fwd	TATAGAATTCGCCACCATGGCTGCAGGAG
MyD88 AgeI Rev	TATAACCGGTGACTCAGGGCAGGGA
MyD88 colonyPCR Fwd	CTGCAGGAGGTCCCGG
MyD88 colonyPCR Rev	GGGCAGGGACAAGGCC
