# Supplementary material for: Transcriptome Analysis of Diffuse Large B-Cell Lymphoma Cells Inducibly Expressing MyD88 L265P Mutation Identifies Upregulated CD44, LGALS3, NFKBIZ, and BATF as Downstream Targets of Oncogenic NF-κB Signaling
Source: Int J Mol Sci. 2023 Mar 15;24(6):5623. doi: 10.3390/ijms24065623 (PMC10057398; doi:10.3390/ijms24065623)
Supplement: Supplementary file 1 [file ijms-24-05623-s001.zip › Table S6 List of primers used for qPCR.pdf]

Supplementary Table S6. List of primers used for qPCR.

Gene name	NCBI accession number	Primer name	Sequence (5' -> 3')	Exon number	Tm (°C)
HPRT	NM_000194	HPRT Fwd	AGCCAGACTTTGTTGGATTTG	7-8	56
		HPRT Rev	TTTACTGGCGATGTCAATAGG	9	55
BATF	NM_006399	BATF Fwd	CCCCTGGCAAACAGGACTCAT	1-2	61
		BATF Rev	GCTTGATCTCCTTGCGTAGAGCC	3	61
LGALS3	NM_002306	LGALS3 Fwd	CAATTTTTCGCTCCATGATGCGTTAT	2-3	59
		LGALS3 Rev	CGGGATAAGCTCCAGGTGCT	3	61
CD44	NM_000610	CD44 Fwd	GAGCCTGGCGCAGATCG	1	62
		CD44 Rev	CTGCAGGTCTCAAATCCGATG	2	58
NFKBIZ	NM_031419	NFKBIZ Fwd	TGTGGATGATTTTAAGACACAAGGTGTGAA	2-3	59
		NFKBIZ Rev	TCTTCCATGCTCTCCCCGG	5	61

RAB29	NM_001135662	RAB29 Fwd	CACGGTGGGAGTGGATTTTGC	2-3	60
		RAB29 Rev	GGACAGATCACACTTGTTGGC	4-5	59
ELL2	NM_012081	ELL2 Fwd	CCATATGTAGGGAAAAGAGTGCAAATT	4-5	59
		ELL2 Rev	TCAGATTGGCTACCTGTTGCAG	5-6	60
TTLL3	NM_001025930	TTLL3 Fwd	AGAGAGCTGTCAAGCAGAAGAAGA	2-3	60
		TTLL3 Rev	CTCATCTTCATCCTCATCCTCAGT	3-4	61
FHIP2B	NM_022749	FHIP2B Fwd	CGCCTACATCCTGGAAGGTAAAAA	5-6	61
		FHIP2B Rev	CCCGACTCTTCTTGCTCTGG	6-7	59
MyD88	NM_002468	MyD88 Fwd	CCAGCATTGAGGAGGATTGCCA	1-2	60
		MyD88 Rev	TCATCTCCTGCACAAACTGGATGT	3	60
